# Multifaceted investigation underlies diverse mechanisms contributing to the downregulation of Hedgehog pathway-associated genes *INTU* and *IFT88* in lung adenocarcinoma and uterine corpus endometrial carcinoma

**DOI:** 10.18632/aging.204262

**Published:** 2022-09-07

**Authors:** Ho Yin Edwin Chan, Zhefan Stephen Chen

**Affiliations:** 1 School of Life Sciences, Faculty of Science, The Chinese University of Hong Kong, Hong Kong SAR, China

**Keywords:** DNA methylation, genomic alteration, Hedgehog pathway, non-coding RNAs, transcription factor

## Abstract

Hedgehog (Hh) signaling primarily functions in the control of mammalian embryonic development but also has roles in cancer. The Hh activation depends on ciliogenesis, a cellular process that describes outgrowth of the primary cilium from cell membrane. Ciliogenesis initiation requires a set of proteins known as planar cell polarity (PCP) effectors. Inturned (INTU) is a PCP effector that reportedly functions synergistically with Hh signaling in basal cell carcinoma, suggesting that INTU has an oncogenic role. In this study, we carried out a pan-cancer investigation on the prognostic significance of *INTU* in different types of cancer. We demonstrated that *INTU* downregulation correlated with reduced survival probabilities in lung adenocarcinoma (LUAD) and uterine corpus endometrial carcinoma (UCEC) patients. Similar expression patterns and prognostic values were identified for *intraflagellar transport 88* (*IFT88*), another Hh pathway-associated gene. We elucidated multiple mechanisms at transcriptional, post-transcriptional and translational levels that involved transcription factor 4 and non-coding RNAs-associated regulatory networks contributing to the reduction of *INTU* and *IFT88* levels in LUAD and UCEC samples. Taken together, this study demonstrates the prognostic significance of the Hh-related genes *INTU* and *IFT88* in LUAD and UCEC and further delineates multifaceted mechanisms leading to *INTU* and *IFT88* downregulation in tumor samples.

## INTRODUCTION

The Hedgehog (Hh) pathway is an evolutionarily conserved signaling axis essential for the regulation of diverse fundamental biological processes, including embryogenesis and tissue homeostasis [1]. Four major components, including Hh ligands, the Patched (PTCH) receptor, the Smoothened (SMO) intermediator and the zinc finger-containing Glioblastoma (GLI) transcription factor, are crucial for mediating signal transduction from the cell membrane to the nucleus. In the absence of Hh ligands, SMO function is inactivated by the PTCH receptor. GLI is converted to the repressor form, which blocks gene transcription. Upon the binding of Hh ligands to the PTCH receptor, SMO inhibition is relieved, leading to the nuclear accumulation of GLI and subsequent activation of Hh target genes [2].

The Hh pathway is crucial for mammalian embryonic development. Activation of the Hh pathway depends on the presence of a specialized cellular organelle known as the primary cilium, where the active SMO protein resides to promote the nuclear translocation of GLI proteins [3]. Normal ciliogenesis relies on a group of planar cell polarity (PCP) effector proteins, including the fuzzy planar cell polarity protein (FUZ), the inturned planar cell polarity protein (INTU) and the WD repeat containing planar cell polarity effector (WDPCP) [4, 5]. The intraflagellar transport (IFT) machinery governs the designated distribution of cargo proteins alongside the ciliary axoneme in support of ciliogenesis and activation of Hh signaling [6]. IFT-A and IFT-B are two subsets of protein complexes necessary for controlling retrograde and anterograde trafficking of cargo proteins [7]. The PCP effectors are indispensable for the initial ciliary recruitment and subsequent transport of IFT-A proteins. In mammalian embryos lacking these essential PCP effector genes, both IFT-A and IFT-B trafficking are impaired. In turn, failure of Hh signaling occurs due to ciliogenesis defects, and this leads to severe developmental retardation and early embryonic mortality [4, 5, 8].

Recently, emerging evidence has emphasized the involvement of the Hh pathway in human age-related disorders and cancers [9]. Oncogenic functions have been assigned to all PCP effectors [10–12]. Interestingly, INTU function was found to be related to the Hh pathway during carcinogenesis [12]. In basal cell carcinoma (BCC), *INTU* expression was aberrantly upregulated, accompanied by the induction of Hh signaling. The disruption of *INTU* in a BCC mouse model ameliorated tumorigenesis, and Hh activation was simultaneously suppressed [12]. Moreover, INTU was found to be functionally upstream of GLI transcription factors. Depletion of *INTU* attenuated the expression of *GLI1* and blocked activation of Hh. However, overexpression of a constitutively activated GLI protein was capable of restoring Hh pathway activity in *INTU*-deficient cells [12]. These findings therefore suggest that INTU plays an important oncogenic function in BCC and highlight a synergistic mechanism involving INTU and Hh signaling in carcinogenesis. To date, the involvement of INTU in other cancer types remains elusive.

In this study, we carried out a comprehensive examination on the prognostic values of *INTU* in 21 different types of cancer. We found that the downregulation of *INTU* was associated with poor prognosis in lung adenocarcinoma (LUAD) and uterine corpus endometrial carcinoma (UCEC) patients. A group of Hh pathway-related genes, including *INTU* and *intraflagellar transport 88* (*IFT88*), were enriched in LUAD and UCEC tumor samples. We further demonstrated positive correlations between *INTU* and *IFT88* levels in both LUAD and UCEC samples, and identified multiple mechanisms spanning transcriptional, post-transcriptional and translational aspects that contribute to *INTU* and *IFT88* downregulation in LUAD and UCEC samples. Taken together, we investigated at multifaceted levels the underlying mechanisms leading to the downregulation of Hh-related genes *INTU* and *IFT88*, and further highlighted the prognostic significance of this downregulation in LUAD and UCEC patients.

## RESULTS

### Investigations on the prognostic values of *INTU* in multiple cancer types

**Figure 1:**
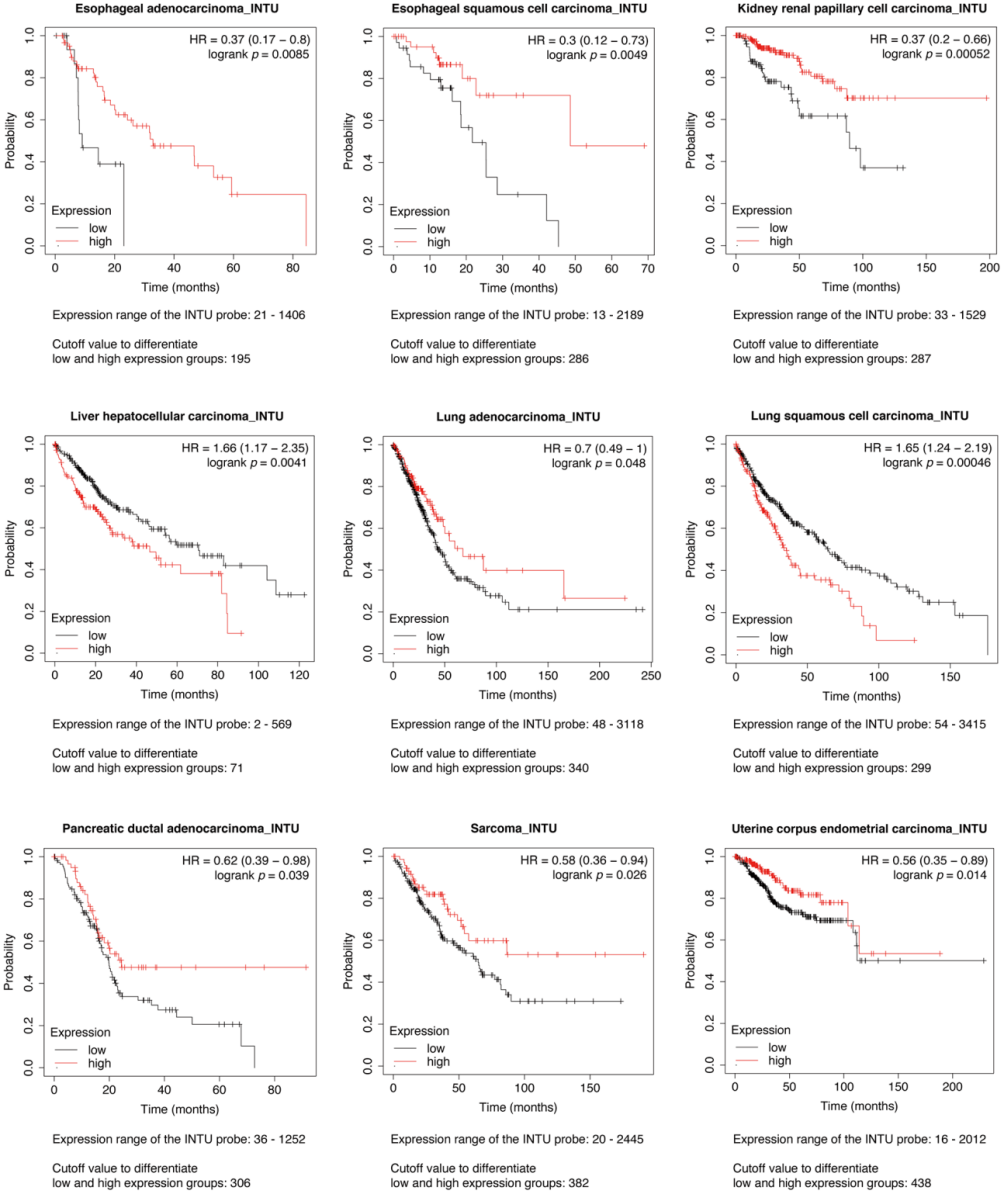
**Evaluation of the prognostic significance of *INTU* mRNA level in different cancer types.** Pan-cancer survival analysis was carried out to determine the relationship between *INTU* mRNA level and OS probabilities in 21 different types of cancer. Decreased *INTU* expression was found associated with poor prognosis in EAC, ESCC, KIRP, LUAD, PDAC, SARC and UCEC patients, whilst high level of *INTU* correlated with poor prognosis in LIHC and LUSC patients.

The Kaplan-Meier plotter was initially used to assess the prognostic significance of *INTU* expression in 21 types of cancer. We found that *INTU* expression was significantly associated with the overall survival (OS) probabilities in patients with nine different cancer types. In liver hepatocellular carcinoma (LIHC) and lung squamous cell carcinoma (LUSC), patients with an increased level of *INTU* had poor OS probabilities. However, in the remaining cancer types, including esophageal adenocarcinoma (EAC), esophageal squamous cell carcinoma (ESCC), kidney renal papillary cell carcinoma (KIRP), LUAD, pancreatic ductal adenocarcinoma (PDAC), sarcoma (SARC) and UCEC, reduced levels of *INTU* were correlated with decreased OS probabilities in patients (Figure 1).

### Hh pathway-related genes were enriched in LUAD and UCEC tumor samples

**Figure 2:**
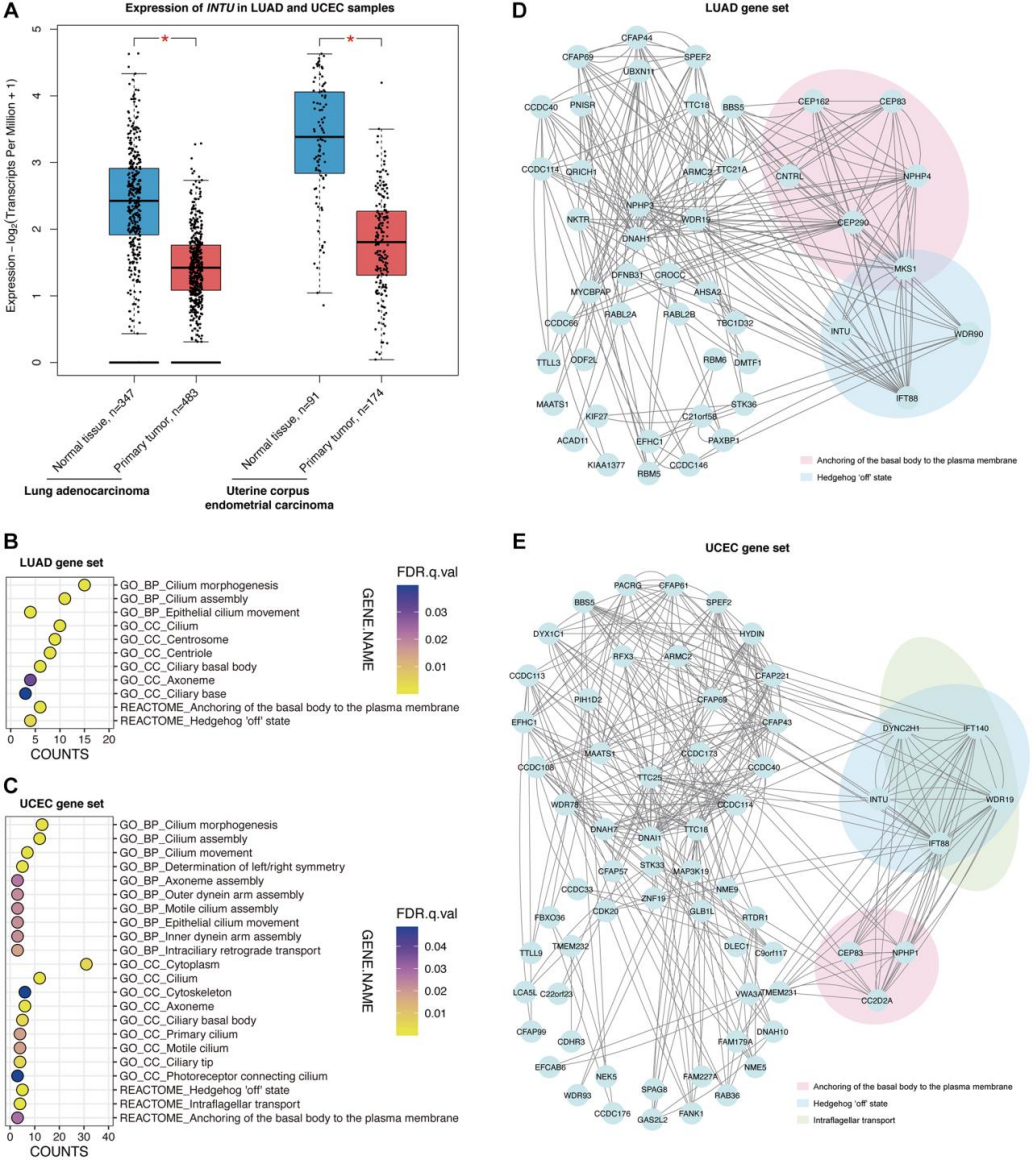
**The Hh pathway-associated genes were enriched in LUAD and UCEC tumor samples.** (**A**) The expression of *INTU* was significantly downregulated in LUAD and UCEC tumor samples. ^*^ denotes *p* < 0.05. (**B**) The GO and Reactome enrichment analysis on the top 100 genes that showed similar expression pattern as *INTU* from LUAD tumor samples. BP indicates biological process, and CC indicates cellular compartment. (**C**) The GO and Reactome enrichment analysis on the top 100 genes that showed similar expression pattern as *INTU* from UCEC tumor samples. (**D**) Construction of the PPI network using genes that showed similar expression pattern as *INTU* in LUAD tumor samples. Two enriched Reactome pathways “Anchoring of the basal body to the plasma membrane” and “Hedgehog ‘off’ state” were highlighted. (**E**) Construction of the PPI network using genes that showed similar expression pattern as *INTU* in UCEC tumor samples. Three enriched Reactome pathways “Anchoring of the basal body to the plasma membrane”, “Hedgehog ‘off’ state” and “Intraflagellar transport” were highlighted.

We next sought to evaluate the expression of *INTU* in tumor samples from different cancer types. The expression of *INTU* in tumor samples from The Cancer Genome Atlas (TCGA) were compared with that from normal samples from TCGA and The Genotype-Tissue Expression project (GTEx). The results showed that the *INTU* transcription level was significantly downregulated in LUAD, LUSC and UCEC samples, whereas in esophageal carcinoma, KIRP, LIHC and SARC, no significant change of *INTU* expression between tumor and normal samples was detected (Figure 2A and Supplementary Figure 1A). No significant changes in the expression of housekeeping genes, including *beta-actin* (*ACTB*), *beta-2-microglobulin* (*B2M*) and *ubiquitin C* (*UBC*), were detected in LUAD and UCEC tumor samples when compared to their respective normal samples (Supplementary Figure 1B). In line with our prognostic analysis, the lower levels of *INTU* in LUAD and UCEC tumor samples (Figure 2A) coincided with poor OS probabilities in cancer patients (Figure 1). We thus decided to focus on LUAD and UCEC in our subsequent studies.

The Gene Expression Profiling Interactive Analysis 2 (GEPIA2) database was used to select the top 100 genes with similar expression patterns as *INTU* from LUAD and UCEC tumor samples (Supplementary Table 1). We then performed gene enrichment analysis to investigate whether certain enriched Gene Ontology (GO) terms and Reactome pathways could be identified from the LUAD and UCEC gene sets. We found that in LUAD, genes were enriched in cilium-associated biological processes, including cilium morphogenesis, assembly and movement. The most enriched subcellular localization pattern was found in association with ciliary compartments. Meanwhile, two Reactome pathways, “Anchoring of the basal body to the plasma membrane” and “Hedgehog ‘off’ state,” were highlighted (Figure 2B). Similar enriched biological processes and cellular compartment terms were identified from the UCEC gene set, where genes were found enriched in three Reactome pathways, including the “Hedgehog ‘off’ state”, “Intraflagellar transport” and “Anchoring of the basal body to the plasma membrane” pathways (Figure 2C).

Protein–protein interaction networks were subsequently constructed. In the LUAD gene set, the centrosomal genes *centrosomal protein 83* (*CEP83*), *centrosomal protein 162* (*CEP162*), *centrosomal protein 290* (*CEP290*), *centriolin* (*CNTRL*), *nephrocystin 4* (*NPHP4*) and *MKS transition zone complex subunit 1* (*MKS1*) were associated with the “Anchoring of the basal body to the plasma membrane” pathway, while *INTU*, *IFT88*, *MKS1* and *WD repeat domain 90* (*WDR90*) were involved in the “Hedgehog ‘off’ state” pathway (Figure 2D). In the UCEC gene set, *coiled-coil and C2 domain containing 2A* (*CC2D2A*), *CEP83* and *nephrocystin 1* (*NPHP1*) were found in the “Anchoring of the basal body to the plasma membrane” pathway. The protein transport-associated genes *dynein cytoplasmic 2 heavy chain 1* (*DYNC2H1*), *IFT88*, *intraflagellar transport 140* (*IFT140*) and *WD repeat domain 19* (*WDR19*) were involved in the “Intraflagellar transport” pathway, while *DYNC2H1*, *INTU*, *IFT88*, *IFT140* and *WDR19* were involved in the “Hedgehog ‘off’ state” pathway (Figure 2E).

### *IFT88* downregulation was associated with the poor prognosis of LUAD and UCEC patients

**Figure 3:**
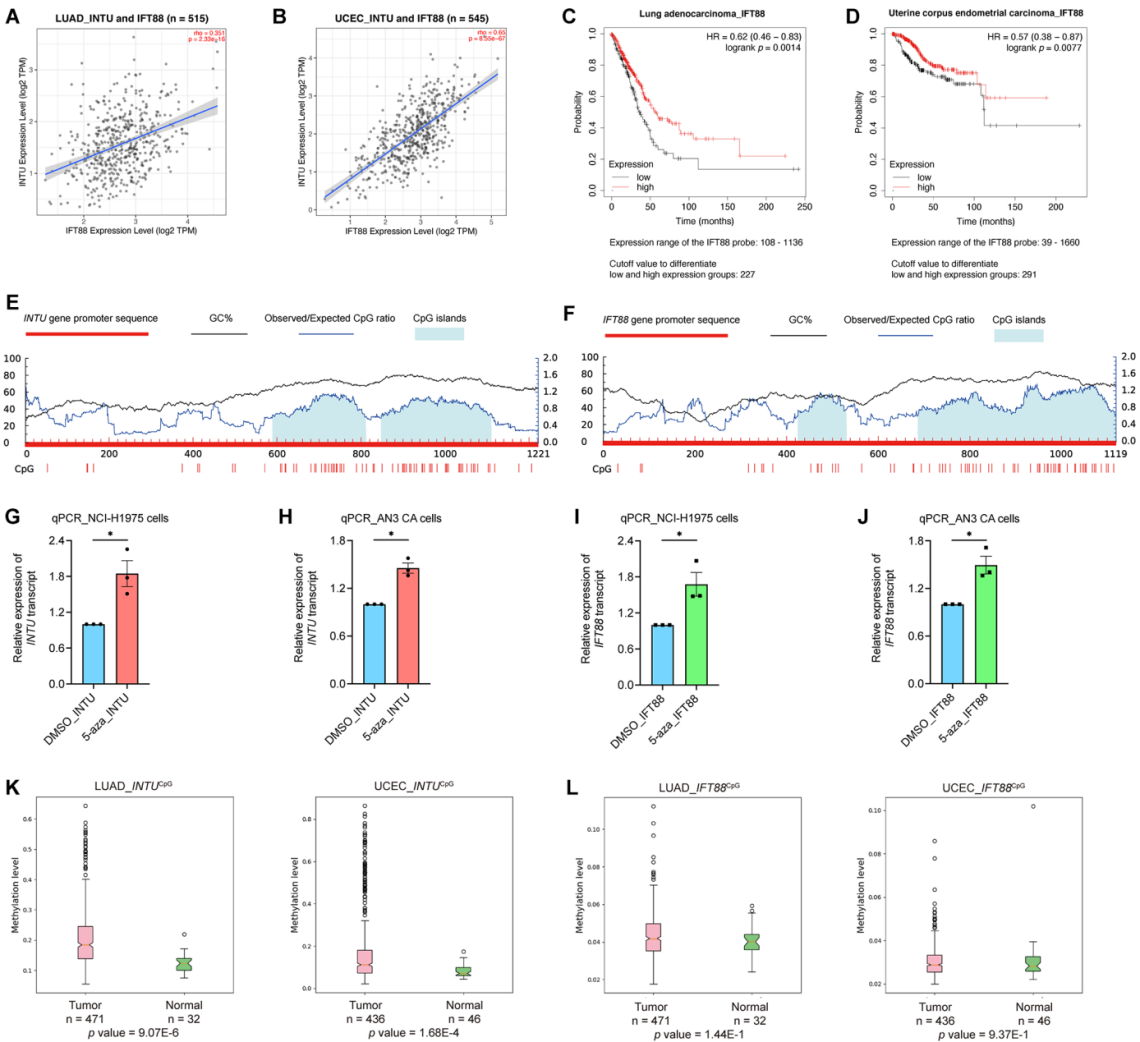
**The expression of *IFT88* correlated with *INTU* expression in LUAD and UCEC samples, and hypermethylation of *INTU*^CpG^ was detected in LUAD and UCEC samples.** (**A**, **B**) The expression of *IFT88* positively correlated with the expression of *INTU* in LUAD (**A**) and UCEC (**B**) tumor samples. (**C**, **D**) Decreased expression of *IFT88* was found associated with reduced OS probabilities in LUAD (**C**) and UCEC (**D**) patients. (**E**) Two putative CpG islands were identified in *INTU* gene promoter. (**F**) Two CpG islands were predicted in *IFT88* gene promoter. (**G**, **H**) Treatment of 5-azacytidine induced *INTU* expression in NCI-H1975 (**G**) and AN3 CA (**H**) cells. *n* = 3 biological replicates. Each *n* represents an independent preparation of cell RNA samples. Error bars represent S.E.M. Statistical analysis was performed using two-tailed unpaired Student’s *t*-test. ^*^ denotes *p* < 0.05. (**I**, **J**) Treatment of 5-azacytidine induced *IFT88* expression in NCI-H1975 (**I**) and AN3 CA (**J**) cells. *n* = 3 biological replicates. Each *n* represents an independent preparation of cell RNA samples. Error bars represent S.E.M. Statistical analysis was performed using two-tailed unpaired Student’s *t*-test. ^*^ denotes *p* < 0.05. (**K**) Hypermethylation of the *INTU*^CpG^ was detected in LUAD and UCEC tumor samples. (**L**) No difference in *IFT88*^CpG^ methylation levels were detected in LUAD and UCEC samples when compared to their respective normal control samples.

Similar to *INTU*, *IFT88* was implicated in the “Hedgehog ‘off’ state” pathway in both the LUAD and UCEC gene sets (Figure 2D, 2E). In addition, *INTU* expression significantly correlated with the expression of *IFT88* from LUAD and UCEC tumor samples (Figure 3A, 3B). Similar to *INTU* (Figure 1), we found that LUAD and UCEC patients with lowered *IFT88* levels also showed decreased OS probabilities (Figure 3C, 3D). Taken together, these results highlight the significance of the downregulation of the Hh pathway-associated genes *INTU* and *IFT88* in the prognosis of LUAD and UCEC patients. We therefore sought to delineate the underlying mechanisms of *INTU* and *IFT88* downregulation in LUAD and UCEC tumor samples.

### The methylation level of *INTU* gene promoter CpG islands was upregulated in LUAD and UCEC tumor samples

DNA methylation is a typical epigenetic modification through which gene expression is modulated [13]. In gene promoter region, CpG islands are regions with densely-accumulated CG dinucleotides, and methylation of CpG islands leads to gene silencing [14]. In each of the *INTU* and *IFT88* gene promoter regions, two putative CpG islands were identified (Figure 3E, 3F). To investigate the association between DNA methylation and *INTU* and *IFT88* gene expression, the LUAD (NCI-H1975) and UCEC (AN3 CA) cell lines were treated with 5-azacytidine, a DNA methyltransferase inhibitor [15], the *INTU* and *IFT88* levels were subsequently detected. We found that upon treatment of 5-azacytidine, both *INTU* and *IFT88* levels were upregulated in NCI-H1975 and AN3 CA cells, suggesting a negative correlation between DNA methylation and *INTU* and *IFT88* gene expression (Figure 3G–3J). The methylation levels of *INTU*^CpG^ and *IFT88*^CpG^ were further examined in LUAD and UCEC tumor samples. We found that the methylation level of *INTU*^CpG^ was significantly upregulated in both LUAD and UCEC tumor samples (Figure 3K). When compared with normal tissues, no significant change in *IFT88*^CpG^ methylation levels was detected in LUAD and UCEC tumor samples (Figure 3L). This suggests that hypermethylation of *INTU*^CpG^ potentially contributes to the reduction of *INTU* levels in LUAD and UCEC samples.

### Involvement of the transcriptional factor TCF4 in the modulation of *INTU* and *IFT88* levels

**Figure 4:**
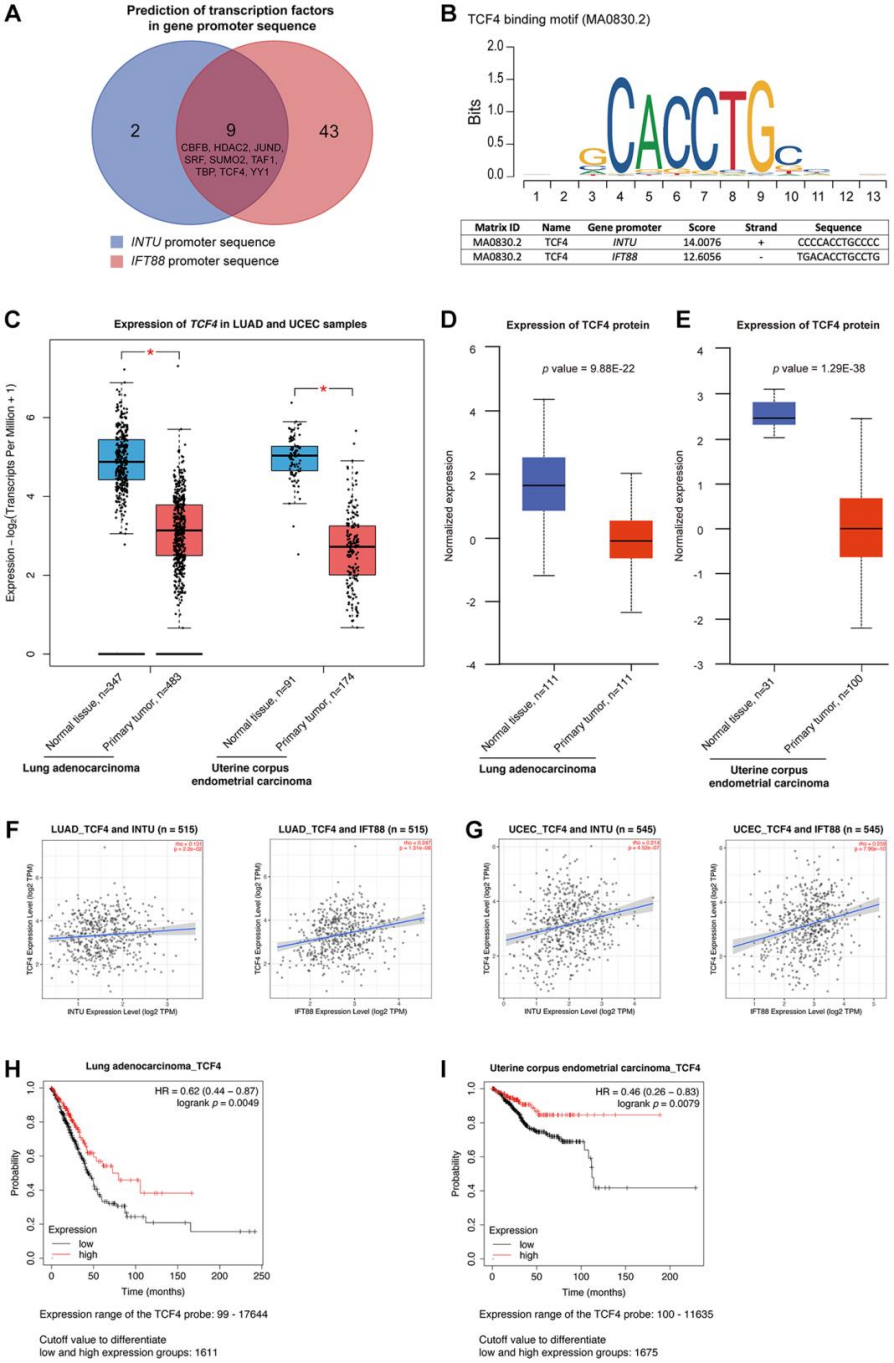
**TCF4 was identified as a potential transcription factor that mediates *INTU* and *IFT88* downregulation in LUAD and UCEC tumor samples.** (**A**) Nine common transcription factors, including CBFB, HDAC2, JUND, SRF, SUMO2, TAF1, TBP, TCF4 and YY1 were predicted in *INTU* and *IFT88* gene promoters. (**B**) Illustration of TCF4 binding consensus sequence and the putative TCF4 binding sites in *INTU* and *IFT88* promoter sequence. (**C**) The *TCF4* transcript level was downregulated in LUAD and UCEC tumor samples. ^*^ denotes *p* < 0.05. (**D**, **E**) The protein level of TCF4 was downregulated in LUAD (**D**) and UCEC (**E**) tumor samples. (**F**, **G**) The expression of *TCF4* positively correlated with the expression of *INTU* and *IFT88* in LUAD (**F**) and UCEC (**G**) tumor samples. (**H**, **I**) The LUAD (**H**) and UCEC (**I**) patients with lowered level of *TCF4* showed reduced OS probabilities.

Transcription factors are a set of regulatory proteins that bind to gene promoter DNA sequences and modulate gene transcription [16]. A total of nine communal transcription factors, including core-binding factor subunit beta (CBFB), histone deacetylase 2 (HDAC2), transcription factor jun-D (JUND), serum response factor (SRF), small ubiquitin-like modifier 2 (SUMO2), TATA-box binding protein associated factor 1 (TAF1), TATA box binding protein (TBP), transcription factor 4 (TCF4) and yin yang 1 (YY1), were identified in the *INTU* and *IFT88* gene promoters (Figure 4A). Next, we evaluated the expression of these transcription factors in LUAD and UCEC samples. Among these nine transcription factors, we found that only *TCF4* (Figure 4B) expression was significantly downregulated in both LUAD and UCEC tumor samples (Figure 4C and Supplementary Figure 2). The TCF4 protein level was further found to be significantly reduced in both LUAD (Figure 4D) and UCEC (Figure 4E) samples. More importantly, the expression of *TCF4* was positively correlated with the expression of *INTU* and *IFT88* in LUAD and UCEC samples (Figure 4F, 4G). Similar to *INTU* (Figure 1) and *IFT88* (Figure 3C, 3D), the reduced levels of *TCF4* were associated with decreased OS probabilities in LUAD and UCEC patients (Figure 4H, 4I). Altogether, TCF4 was identified as a putative upstream regulator in controlling the expression of *INTU* and *IFT88* in LUAD and UCEC tumor samples.

**Figure 5:**
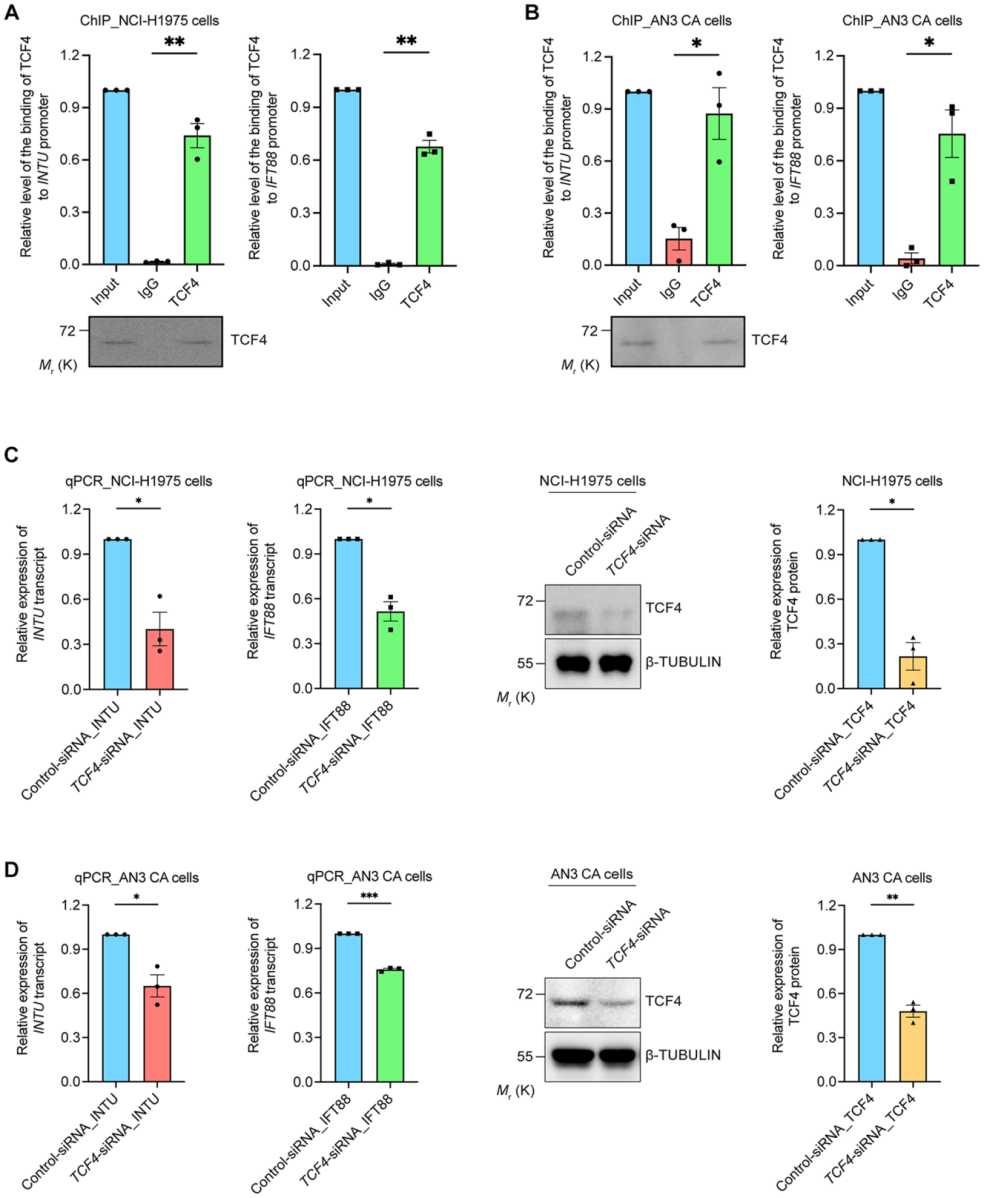
**TCF4 interacted with *INTU* and *IFT88* promoters and mediated their gene expression.** (**A**, **B**) The binding between TCF4 and *INTU* and *IFT88* promoters was detected in NCI-H1975 (**A**) and AN3 CA (**B**) cells. *n* = 3 biological replicates. Each *n* represents an independent preparation of ChIP samples. Error bars represent S.E.M. Statistical analysis was performed using two-tailed unpaired Student’s *t*-test. ^*^ denotes *p* < 0.05 and ^**^ denotes *p* < 0.01. (**C**, **D**) Knockdown of *TCF4* downregulated the transcript levels of *INTU* and *IFT88* in NCI-H1975 (**C**) and AN3 CA (**D**) cells. *n* = 3 biological replicates. Each *n* represents an independent preparation of RNA and protein samples. Error bars represent S.E.M. Statistical analysis was performed using two-tailed unpaired Student’s *t*-test. ^*^denotes *p* < 0.05, ^**^denotes *p* < 0.01 and ^***^denotes *p* < 0.001.

We further provided experimental evidence in support of our findings. Chromatin immunoprecipitation was performed to investigate the interaction between TCF4 protein and *INTU* and *IFT88* gene promoters. We found that in both the NCI-H1975 and AN3 CA cells, the binding of TCF4 to *INTU* and *IFT88* gene promoters was detected (Figure 5A, 5B). The transcriptional regulatory function of TCF4 on *INTU* and *IFT88* gene expression was subsequently determined. When endogenous *TCF4* was knocked down in NCI-H1975 and AN3 CA cells, the transcript levels of *INTU* and *IFT88* were downregulated (Figure 5C, 5D). These data further support the positive correlation between *TCF4* and *INTU* and *IFT88* levels in LUAD and UCEC samples (Figure 4F, 4G).

### Identification of *hsa-miR-212-3p* as the upstream microRNA targeting Hh-related genes *INTU* and *IFT88*

**Figure 6:**
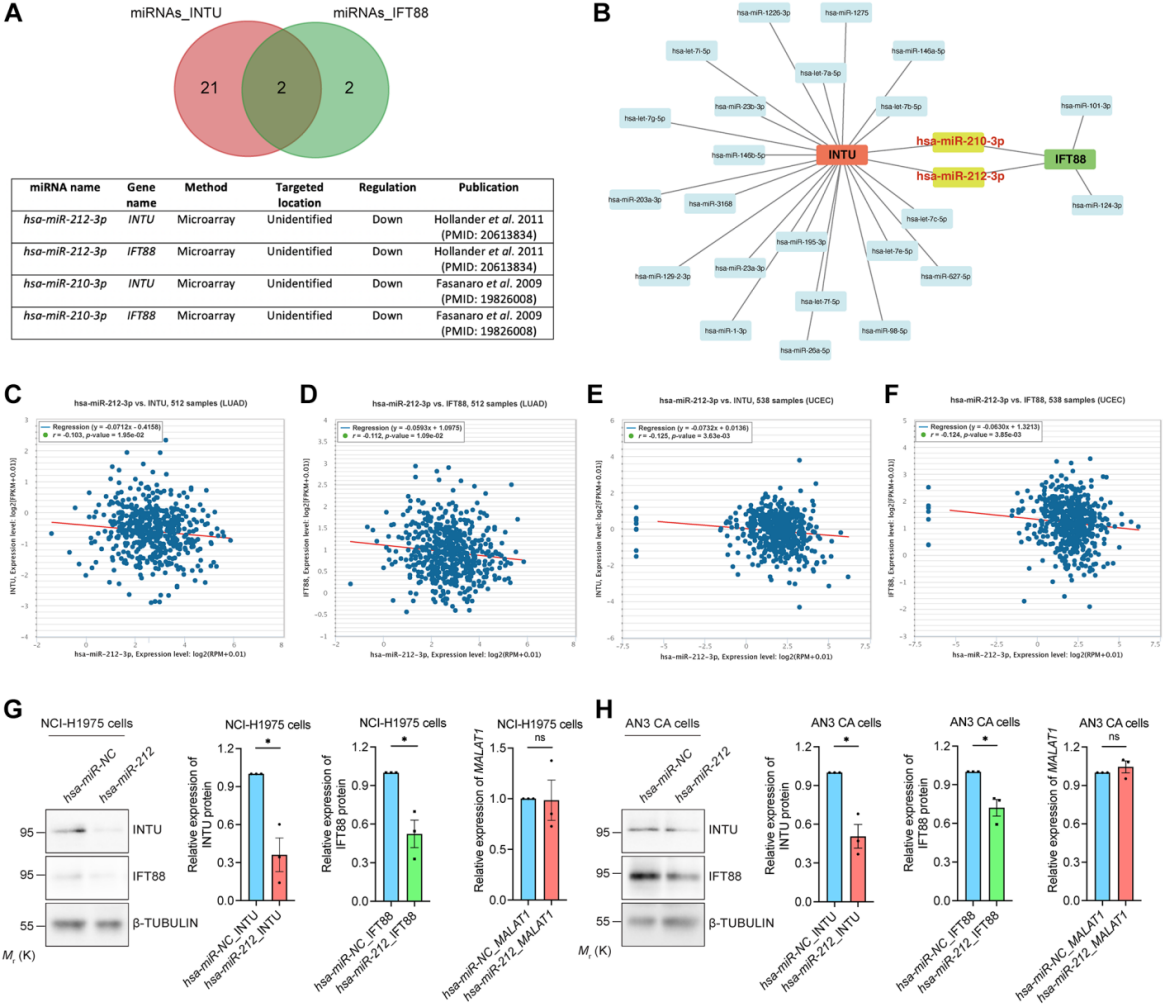
**Identification of *hsa-miR-212-3p* as a communal miRNA against *INTU* and *IFT88* in LUAD and UCEC samples.** (**A**) Identification of *hsa-miR-212-3p* and *hsa-miR-210-3p* miRNAs that target both *INTU* and *IFT88* transcripts. (**B**) Construction of the miRNA-target gene regulatory network. (**C**, **D**) The expression of *hsa-miR-212-3p* negatively correlated with the expression of *INTU* (**C**) and *IFT88* (**D**) in LUAD samples. (**E**, **F**) The expression of *hsa-miR-212-3p* negatively correlated with the expression of *INTU* (**E**) and *IFT88* (**F**) in UCEC samples. (**G**, **H**) Overexpression of *hsa-miR-212* led to the downregulation of INTU and IFT88 protein levels in NCI-H1975 (**G**) and AN3 CA (**H**) cells. The *MALAT1* levels were not affected. *n* = 3 biological replicates. Each *n* represents an independent preparation of protein or RNA samples. Error bars represent S.E.M. Statistical analysis was performed using two-tailed unpaired Student’s *t*-test. ns indicates no significant difference. ^*^ denotes *p* < 0.05.

In addition to gene silencing, post-transcriptional regulation of gene expression mediated by non-coding RNAs (ncRNAs), including microRNAs (miRNAs) and long non-coding RNAs (lncRNAs), has been reported [17, 18]. The DIANA-TarBase v8 database was used to select potential miRNAs that target *INTU* or *IFT88* based on experimental evidence [19]. We identified 23 miRNAs that target the *INTU* transcript and four miRNAs that target the *IFT88* transcript (Figure 6A). Interestingly, two miRNAs, *hsa-miR-210-3p* [20] and *hsa-miR-212-3p* [21], were shown to target both *INTU* and *IFT88* (Figure 6A, 6B). We next evaluated the correlation between the miRNA level and *INTU* or *IFT88* expression in LUAD and UCEC tumor samples. The expression of *hsa-miR-212-3p*, but not *hsa-miR-210-3p*, was found to be negatively correlated with the expression of *INTU* and *IFT88* in LUAD and UCEC samples (Figure 6C–6F and Supplementary Figure 3). Moreover, we found that when *hsa-miR-212* was overexpressed in NCI-H1975 and AN3 CA cells, downregulation of INTU and IFT88 protein levels were detected (Figure 6G, 6H). This further suggests the negative regulatory function of *hsa-miR-212* on the expression of INTU and IFT88.

### Identification of *MALAT1* as an upstream lncRNA

**Figure 7:**
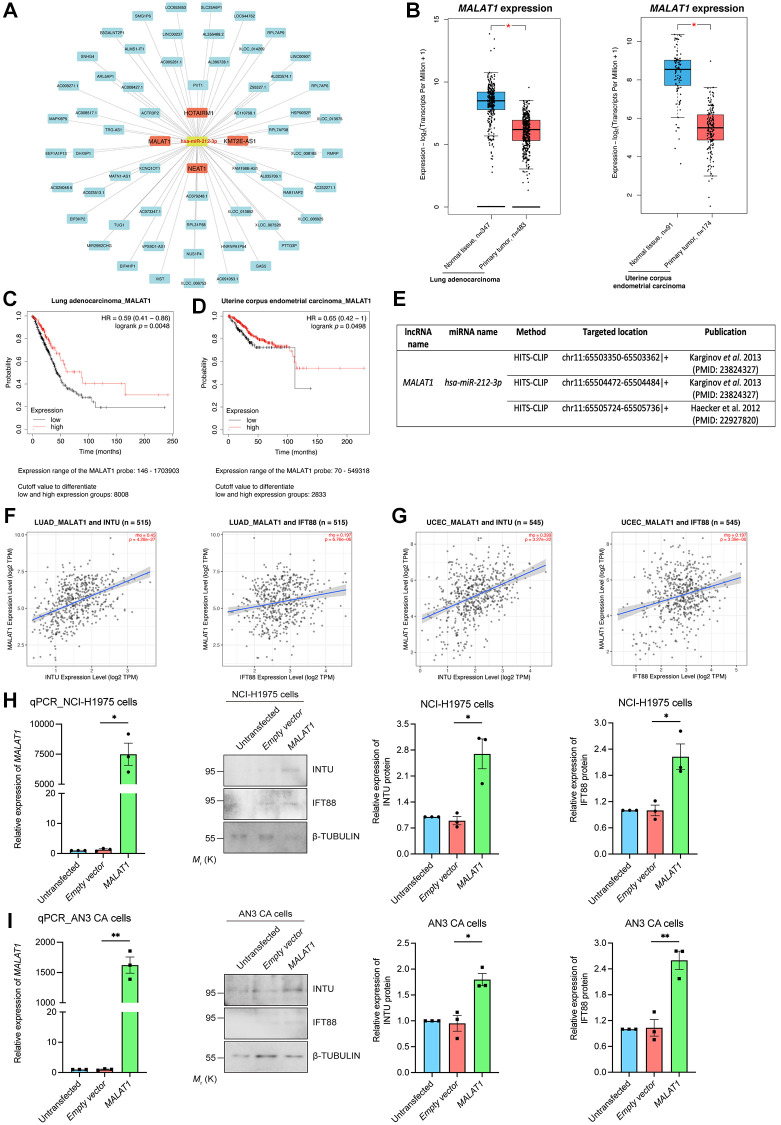
**Identification of *MALAT1* as a communal lncRNA mediating *INTU* and *IFT88* expression in LUAD and UCEC samples.** (**A**) Identification of the lncRNAs against *hsa-miR-212-3p* and construction of the lncRNA-miRNA regulatory network. (**B**) The expression of *MALAT1* was significantly downregulated in LUAD and UCEC tumor samples. (**C**, **D**) Decreased level of *MALAT1* was found associated with poor prognosis in LUAD (**C**) and UCEC (**D**) patients. (**E**) The *MALAT1* was identified as targeted lncRNA against *hsa-miR-212-3p* from two independent studies. (**F**, **G**) The expression of *MALAT1* positively associated with the expression of *INTU* and *IFT88* in LUAD (**F**) and UCEC (**G**) tumor samples. (**H**, **I**) Overexpression of *MALAT1* caused the upregulation of INTU and IFT88 protein levels in NCI-H1975 (**H**) and AN3 CA (**I**) cells. *n* = 3 biological replicates. Each *n* represents an independent preparation of RNA and protein samples. Error bars represent S.E.M. Statistical analysis was performed using two-tailed unpaired Student’s *t*-test. ^*^ denotes *p* < 0.05.

We next examined upstream lncRNAs using the DIANA-LncBase v3 database. A total of 63 lncRNAs were obtained, and their expression levels in LUAD and UCEC samples were evaluated (Figure 7A). The levels of four of the 63 lncRNAs, including *HOXA transcript antisense RNA, myeloid-specific 1* (*HOTAIRM1*), *KMT2E antisense RNA 1* (*KMT2E-AS1*), *metastasis associated lung adenocarcinoma transcript 1* (*MALAT1*) and *nuclear paraspeckle assembly transcript 1* (*NEAT1*), were significantly downregulated in LUAD and UCEC tumor samples when compared with their respective normal tissues (Figure 7B and Supplementary Figure 4A). The prognostic significance of these four lncRNAs were further evaluated. We found that *MALAT1*, but not *HOTAIRM1* and *NEAT1*, showed prognostic significance in both LUAD and UCEC patients (Figure 7C, 7D and Supplementary Figure 4B). Similar to what was detected regarding *INTU* (Figure 1) and *IFT88* (Figure 3C, 3D), the reduced level of *MALAT1* contributed to poor OS probabilities in LUAD and UCEC patients (Figure 7C, 7D). Overexpression of *hsa-miR-212* did not modulate *MALAT1* levels in NCI-H1975 and AN3 CA cells (Figure 6G, 6H). In contrast, *MALAT1* has been shown to target *hsa-miR-212-3p* in two independent studies (Figure 7E) [22, 23]. Moreover, the expression of *MALAT1* was found to be positively associated with the mRNA levels of *INTU* and *IFT88* in LUAD and UCEC tumor samples (Figure 7F, 7G). To further validate the regulatory function of *MALAT1* on the expression of INTU and IFT88, *MALAT1* was overexpressed in NCI-H1975 or AN3 CA cells (Figure 7H, 7I). We found that in *MALAT1*-overexpressing cells, the protein levels of INTU and IFT88 were simultaneously increased (Figure 7H, 7I). These results therefore confirm the role of *MALAT1* in regulating the expression of INTU and IFT88 in lung and endometrial cancer cells.

## DISCUSSION

In this study, we demonstrated that the downregulation of the *INTU* and *IFT88* was correlated with reduced survival probabilities in LUAD and UCEC patients (Figures 1, 3C, 3D). We next sought to explore the driving forces causing this downregulation in LUAD and UCEC tumor samples, and identified multifaceted mechanisms in the DNA, RNA and protein levels contributing to *INTU* and *IFT88* downregulation (Figures 3–7). This study provides a comprehensive mechanistic investigation regarding *INTU* and *IFT88* downregulation in cancer, and further highlights the involvement of Hh signaling in carcinogenesis.

The dysregulation of Hh signaling has been documented in multiple types of cancer. In BCC, medulloblastoma and rhabdomyosarcoma, mutations in Hh-related genes activate Hh signaling in support of the over-proliferation and tissue invasion of cancer cells [24–26]. In addition to genetic mutation, epigenetic modification also contributes to aberrant Hh signaling in tumor samples. For example, the hypermethylation of *hedgehog-interacting protein* (HHIP), a gene encodes for a negative regulator of Hh signaling, was determined in pancreatic cancer samples. This leads to the reduced expression of HHIP, followed by the upregulation of Hh signaling [27]. In the majority of solid tumors, including colorectal cancer (CRC), the mutation in Hh-related genes was rarely detected [28]. Interestingly, in CRC, the stromal Hh pathway targets were found downregulated despite the increased expression of Hh ligand. This might be due to the insensitivity of stromal cells to epithelial Hh ligand, or the impairment of tissue architecture in tumor stroma [29, 30]. In addition, the restoration of stromal Hh signaling markedly alleviated tumorigenesis, whereas inhibition of Hh signaling exacerbated tumor progression [31]. These findings taken together suggest multiple mechanisms in contributing to the dysregulation of Hh signaling in different cancers, and also highlight the oncogenic role of Hh signaling. Here, we reported two subsets of enriched Hh pathway-associated genes with similar downregulation expression patterns in LUAD and UCEC tumor samples (Figure 2D, 2E). We further showed that lowered levels of *INTU*, *IFT88* or *MKS1* were correlated with decreased OS probabilities in LUAD patients, while UCEC patients with reduced *INTU*, *IFT88* or *IFT140* levels had a poor prognosis (Figures 1, 3C, 3D, Supplementary Figure 5A, 5B). INTU was found to be necessary for the ciliary recruitment of IFT-A proteins, and MKS1 functionally associates with IFT complexes in mediating the transport of cargo proteins to support ciliary outgrowth [8, 32]. Depletion of *INTU* or IFT machinery components leads to ciliogenesis defects [12, 33, 34]. Importantly, the loss of ciliary structures has been recorded in lung and endometrial cancer patient samples [35, 36]. Moreover, stimulation of ciliogenesis has been reported to combat against lung cancer cell proliferation, invasion and epithelial-mesenchymal transition [37]. These findings therefore indicate that disruption of Hh signaling components might cause ciliogenesis defects, which in favour of oncogenesis in LUAD and UCEC tissues.

TCF4 belongs to the helix–loop–helix (HLH) family of proteins ubiquitously expressed throughout different human tissues [38]. A basic residue group on the TCF4 N-terminus is critical for its DNA-binding function, while the C-terminal HLH domain mediates the dimerization of TCF4 when it binds to DNA [39]. Several studies have demonstrated the association between TCF4 function and DNA methylation [40, 41]. Specifically, TCF4 was correlated with DNA hypomethylation in mammalian epithelial stem cells. Upon conditional knockout of *TCF4*, the TCF4-bound differentially methylated DNA sequence was found to be strongly methylated [41]. Interestingly, when looking into the TCF4 binding site in the *INTU* promoter sequence, we found that the TCF4 site resides in the *INTU*^CpG^ (highlighted in Supplementary Table 2). The methylation level of *INTU*^CpG^ was upregulated in LUAD and UCEC samples (Figure 3G). Reduction of the TCF4 protein level was recorded in both LUAD and UCEC tumor samples (Figure 4D). Such attenuation of the TCF4 level might result in *INTU*^CpG^ hypermethylation, which in turn leads to the downregulation of *INTU* expression in LUAD and UCEC samples. Meanwhile, TCF4 did not associate with *IFT88*^CpG^ (Supplementary Table 2), and no significant change in *IFT88*^CpG^ methylation level was detected in LUAD and UCEC samples (Figure 3H).

Genetic mutations in *TCF4* have been reported in neurological disorders, including Fuchs’s corneal dystrophy [42], Pitt–Hopkins syndrome [43], and schizophrenia [44], as well as non-neurological diseases, including primary sclerosing cholangitis [45] and sporadic Sonic Hedgehog-associated medulloblastoma (SHH MB) [46]. Functional analysis was carried out on mutant TCF4 proteins harboring the mutations identified from SHH MB patients. Experimental findings highlighted the loss-of-function behind these TCF4 mutations, as exemplified by the fact that mutant TCF4 proteins failed to inhibit the proliferation of medulloblastoma cells, unlike the wild-type TCF4 protein [40, 47]. We also found a nonsense mutation at the arginine 174 residue (R174^*^) on the TCF4 protein in eight UCEC patient samples (Supplementary Figure 6A, 6B), and the R174 site was conserved across different species (Supplementary Figure 6C). This nonsense mutation generates a truncated TCF4 protein which lacks the C-terminal HLH motif that is crucial for mediating gene transcription, suggesting the loss of TCF4 transactivating function due to the presence of such a mutation. This could serve as another mechanism leading to the downregulation of Hh-related genes in UCEC tumor samples. Interestingly, a similar TCF4^R174*^ mutation was previously reported in patients with SHH MB and Pitt–Hopkins syndrome [46, 47], suggesting that the communal loss-of-function mechanism is involved in a broad spectrum of human disorders.

miRNAs and lncRNAs are two major subtypes of ncRNAs associated with the well-documented ceRNA mechanism that is essential for controlling gene expression at a post-transcriptional level [18, 48]. We identified a novel and communal *MALAT1*-*hsa-miR-212-3p* regulatory network that downregulated *INTU* and *IFT88* expression in LUAD and UCEC samples (Figure 5). The recurrent fusion of *MALAT1* with the *GLI1* gene was reported in patients with gastroblastoma and plexiform fibromyxoma [49, 50]. This *MALAT1-GLI1* fusion mutation activated Hh signaling and consequently led to malignant tumor formation, suggesting a relationship between *MALAT1* function and Hh signaling activity [49, 51, 52]. In this study, we further highlighted the involvement of *MALAT1* in regulating Hh pathway-associated genes. We found that in addition to *INTU* and *IFT88*, other Hh-related genes with similar expression profiles as *INTU* were also enriched in LUAD and UCEC samples (Figure 2D, 2E). Interestingly, the expression of *hsa-miR-212-3p* was negatively associated with the expression of *MKS1* and *WDR90* in LUAD samples (Supplementary Figure 7A) and *DYNC2H1*, *IFT140* and *WDR19* in UCEC samples (Supplementary Figure 7B). Positive correlations were determined between *MALAT1*, *MKS1* and *WDR90* in LUAD samples and *MALAT1*, *DYNC2H1* and *WDR19* in UCEC samples (Supplementary Figure 7C, 7D). Taken together, these findings emphasize the *MALAT1*-*hsa-miR-212-3p* network as a master upstream regulator targeting downstream Hh pathway-associated genes in LUAD and UCEC tumor samples. In addition, *MALAT1* binds to active chromatin sites and regulates gene transcription by recruiting chromatin modifiers or transcription regulators to specific genomic loci [53, 54]. This might be an alternative mechanism that accounts for *MALAT1*’s regulation on Hh pathway-associated gene expression and is worthy to be further investigated.

Several recent studies have reported the anti-tumorigenesis role of TCF4 in colorectal carcinoma and SHH MB [47, 55]. TCF4 is capable of attenuating the proliferation of colon cancer and medulloblastoma cells, whereas loss of *TCF4* exerts the opposite effect, favoring tumorigenesis [47, 56, 57]. The effect of *MALAT1* on tumor cell growth and invasion is controversial. Although the oncogenic functions of *MALAT1* have been reported in malignancies such as colorectal and liver cancer [58, 59], more recent studies have highlighted the tumor suppressive role of *MALAT1* against the growth and metastasis of glioma and breast cancer cells [60, 61]. These findings taken together suggest a cancer type-dependent role of *MALAT1* in tumorigenesis. Given the functions of TCF4 and *MALAT1* as tumor suppressors, targeting the functional elevation of TCF4 and *MALAT1* could be therapeutically beneficial against tumorigenesis. In fact, the identification of small molecule drugs aimed at stimulating TCF4 function is now under investigation (Pitt Hopkins Research Foundation; https://pitthopkins.org/portfolio-item/pilot-study-to-identify-small-molecule-activators-of-tcf4-as-a-treatment-for-pitt-hopkins-syndrome/). Meanwhile, different small molecule activators for *MALAT1* have been reported (Table 1). A combinatorial drug treatment has been demonstrated as an effective therapeutic strategy in combating carcinogenesis [62–64]. The use of both TCF4 and *MALAT1* activators in the treatment against LUAD and UCEC would be an interesting topic worthy of further exploration.

**Table 1:** Summary of *MALAT1* small molecule activators.

**lncRNA**	**Small molecules**	**Effect on lncRNA expression**	**Approved by FDA**	**Validated by experiments**	**Validation method**	**Experimental material**	**References**
*MALAT1*	Carboplatin + Docetaxel	Up-regulation	Yes	Yes	Quantitative real-time PCR	Ovarian cancer cell line	[81]
*MALAT1*	Quercetin	Up-regulation	Yes	Yes	Quantitative real-time PCR	Rheumatoid arthritis fibroblast-like synoviocytes	[82]

**Figure 8:**
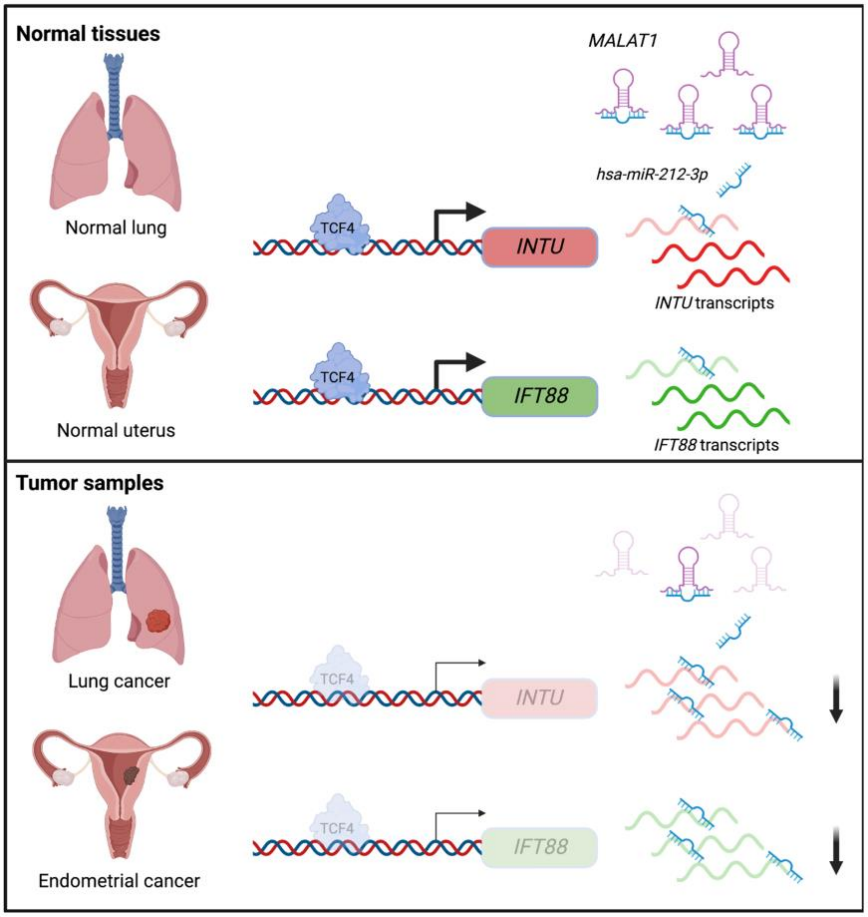
Illustration of the underlying mechanisms that contribute to *INTU* and *IFT88* downregulation in the lung and endometrial cancers.

In summary, we showed that the downregulation of the Hh pathway-associated genes *INTU* and *IFT88* was correlated with poor prognosis in LUAD and UCEC patients. Moreover, we demonstrated novel TCF4 and ncRNA-involved mechanisms that contribute to the downregulation of *INTU* and *IFT88* in LUAD and UCEC tumor samples (Figure 8). We further propose that a treatment strategy that simultaneously targets TCF4 and *MALAT1* to enrich *INTU* and *IFT88* might be a promising therapeutic intervention against LUAD and UCEC.

## MATERIALS AND METHODS

### Kaplan-Meier plotter analysis

The pan-cancer analysis function (https://kmplot.com/analysis/index.php?p=service&cancer=pancancer_rnaseq) from the Kaplan-Meier plotter database was used to evaluate the prognostic significance of *INTU* mRNA expression in 21 different types of cancer [65]. The prognostic significance of Hh pathway-associated genes *IFT88*, *MKS1*, *WDR90*, *IFT140*, *DYNC2H1* and *WDR19* was also evaluated using LUAD and UCEC patient survival data from Kaplan-Meier plotter database. Similar approach was used to determine the prognostic value of TCF4 and lncRNAs. The OS probabilities of cancer patients were assessed using the Kaplan-Meier survival plots, and logrank *p* < 0.05 indicates that the association between gene/lncRNA expression and patient survival probability is statistically significant.

### GEPIA2 database analysis

GEPIA2 (http://gepia2.cancer-pku.cn/#index) is an online database that provides gene expression profiling and interactive analyses in primary tumor and normal tissue samples on the basis of data from The Cancer Genome Atlas (TCGA) and the Genotype-Tissue Expression (GTEx) projects [66]. The expression of *INTU*, housekeeping genes, different transcription factors and different lncRNAs were determined in LUAD and UCEC primary tumor samples and their respective normal tissues. The *p* < 0.05 was considered as statistically significant. The top 100 genes with similar expression pattern as *INTU* in LUAD or UCEC tumor samples were also selected using the “Similar Gene Detection” function from GEPIA2. The detailed gene lists are included in Supplementary Table 1.

### Gene ontology and Reactome pathway analysis

The *INTU* and top 100 genes with similar expression pattern obtained from GEPIA2 database were input to Database for Annotation, Visualization and Integrated Discovery (DAVID) v6.8 (https://david.ncifcrf.gov/home.jsp) to analyze their enriched Gene Ontology (GO) terms and Reactome pathways [67]. The biological processes (BP) and cellular components (CC) were included in the GO enrichment analysis. The false discovery rate (FDR) *q*-value < 0.05 was used as selection criteria for significantly enriched GO terms and Reactome pathways.

### Protein-protein interaction (PPI) network analysis

The construction of PPI network was performed using STRING v11.5 database (https://string-db.org/) [68]. The genes with similar expression pattern obtained from GEPIA2 database were input to STRING database, and the PPI network was constructed based on the sources of “Co-expression”, “Databases”, “Experiments”, “Gene Fusion”, “Neighborhood” and “Textmining” with minimum required interaction score of medium confidence. The Cytoscape v3.8.0 was used to visualize the constructed PPI network [69].

### Prediction of CpG islands and transcription factor binding sites within gene promoter sequences

*INTU* and *IFT88* promoter sequences were withdrawn from GenBank under the accession numbers NC_000004.12 and NC_000013.11, respectively. The CpG islands were predicted using MethPrimer 2.0 (http://www.urogene.org/cgi-bin/methprimer2/MethPrimer.cgi) software [70]. The transcription factor binding sites were predicted using Animal Transcription Factor Database 3.0 (AnimalTFDB3.0; http://bioinfo.life.hust.edu.cn/AnimalTFDB/#!/) [71]. The “*q*-value < 0.05” and “Score > 20” were used as filtering parameters to select potential transcription factors for *INTU* and *IFT88*. The TCF4 binding site was further validated using JASPAR (https://jaspar.genereg.net/) database [72]. The relative profile score threshold equals to 90%, and “Score > 12.5” and “Relative score > 0.92” were used as filtering parameters. The detailed *INTU* and *IFT88* promoter sequences are listed in Supplementary Table 2, the putative CpG islands are shown in blue and the TCF4 binding sites are highlighted.

### MethHC2.0 database analysis

MethHC2.0 is a web-based resource that provides analysis on the methylation levels of gene regions, including CpG islands, from different types of cancer [73]. The methylation levels of *INTU* and *IFT88* CpG islands were scrutinized using methylome data from LUAD and UCEC tumor samples and their respective normal samples.

### UALCAN database analysis

UALCAN (http://ualcan.path.uab.edu/analysis-prot.html) is an interactive online resource that enables the analysis of protein expression based on the Clinical Proteomic Tumor Analysis Consortium Confirmatory/Discovery datasets [74]. In this study, the TCF4 protein expression from LUAD and UCEC tumor samples and their respective normal tissues was analyzed.

### Candidate miRNA and lncRNA prediction

The list of miRNAs that target *INTU* and *IFT88* was obtained using DIANA-Tarbase v8 database (https://dianalab.e-ce.uth.gr/html/diana/web/index.php?r=tarbasev8%2Findex) [19]. The “Species = *Homo Sapiens*” and “Validated as Positive” were used as filtering parameters. The miRNA-target genes regulatory network was constructed using Cytoscape v3.8.0.

The list of lncRNAs that target different miRNAs was obtained using DIANA-LncBase v3 database (https://diana.e-ce.uth.gr/lncbasev3) [75]. The “Species = *Homo Sapiens*”, “miRNA Conf. Level = High” and “Validated as Positive” were used as filtering parameters. The lncRNA-miRNA regulatory network was constructed using Cytoscape v3.8.0.

### ENCORI database analysis

ENCORI (http://starbase.sysu.edu.cn/) is an online database that determines the correlation between miRNA level and target gene expression [76]. The expression correlation between *hsa-miR-210-3p*/*hsa-miR-212-3p* and different enriched Hh-related genes was analyzed using “miRNA-Target CoExpression” module from the “Pan-Cancer” function. The *p*-value < 0.05 was considered as statistically significant.

### TIMER2.0 database analysis

The TIMER2.0 (http://timer.cistrome.org/) is an online web server that enables the detection of gene expression correlation [77]. The correlation among different Hh-related genes, and correlation between Hh-related genes and *MALAT1* in LUAD and UCEC tumor samples were evaluated using the “Gene_Correlation” module. No adjustment was made, and *p* < 0.05 was considered as statistically significant.

### cBioPortal database analysis

cBioPortal v3.7.3 is a comprehensive web resource that enables the visualization and analysis of cancer genomic mutation data (https://www.cbioportal.org/) [78, 79]. The missense and nonsense mutation profiles in *INTU*, *IFT88* and *TCF4* genes were obtained from Lung Adenocarcinoma (TCGA, PanCancer Atlas, 566 samples) and Uterine Corpus Endometrial Carcinoma (TCGA, PanCancer Atlas, 529 samples) datasets.

### D-lnc database

The D-lnc database (http://www.jianglab.cn/D-lnc/index.jsp) is a comprehensive platform that summarizes the lncRNA-targeting drugs based on the experimental evidence and computational predictions [80]. The “Species = *Homo Sapiens*” and “lncRNA = *MALAT1*” were used to select potential small molecules that target *MALAT1*.

### Mammalian cell culture

The human endometrial adenocarcinoma cell line AN3 CA was a kind gift from Prof. Chi Chiu Wang (Department of Obstetrics and Gynecology, The Chinese University of Hong Kong, China). The human lung adenocarcinoma cell line NCI-H1975 (CRL-5908™) was obtained from American Type Culture Collection. Both cell lines were cultured using Dulbecco’s Modified Eagle’s Medium (11995065, Thermo Fisher Scientific) supplemented with 10% fetal bovine serum (F7524, Sigma-Aldrich) and 1% penicillin-streptomycin solution (15140122, Thermo Fisher Scientific). The cells were maintained in a 37°C humidified cell culture incubator supplemented with 5% CO_2_.

### Plasmid, microRNA and siRNA transfection

The *pcDNA-MALAT1* plasmid was a kind gift from Prof. Huating Wang (Department of Orthopaedics and Traumatology, The Chinese University of Hong Kong, China). Cells were transfected with 0.5 μg *pcDNA-MALAT1* plasmid with 0.5 μl lipofectamine 2000 (11668019, Thermo Fisher Scientific). The RNA or protein samples were harvested 24 h post transfection. The *hsa-miR-NC* (4464058) and *hsa-miR-212* (4464066) were synthesized by Thermo Fisher Scientific. Cells were transfected with 20 pmol microRNAs with 2 μl lipoRNAiMAX (13778150, Thermo Fisher Scientific). Cell culture medium and transfection mixtures were refreshed every 24 h, and protein samples were harvested 72 h post transfection. The Control-siRNA, 5′-UUCUCCGAACGUGUCACGUTT-3′ and *TCF4*-siRNA, 5′-CUAUCAGUAUUCUAGCAAUAATT-3′ were synthesized by Sangon Biotech (Shanghai) Co., Ltd. Cells were transfected with 20 pmol siRNAs with 2 μl lipoRNAiMAX. Cell culture medium and transfection mixtures were refreshed every 24 h, and RNA or protein samples were harvested 72 h post transfection.

### Drug treatment

The NCI-H1975 and AN3 CA cells were treated with 2 μM 5-azacytidine (A2385, Sigma-Aldrich). The treatment lasted 72 h, with medium and drug refreshed every 24 h.

### Chromatin immunoprecipitation

The chromatin immunoprecipitation (ChIP) assay was performed using Pierce™ Magnetic ChIP Kit (26157, Thermo Fisher Scientific). The experimental procedures were carried out following the manufacturer’s instructions. Two micrograms of anti-TCF4 antibody (ab217668, abcam) were used for the immunoprecipitation, while the same amount of normal rabbit IgG (I-1000, Vector Laboratories, lnc.) was used as negative control. Twenty nanograms of recovered genomic DNAs from each of input, normal rabbit IgG and TCF4 immunoprecipitated samples were used in the following real-time PCR to analyze the levels of *INTU* and *IFT88* promoter fragments. The primers used were *INTU promoter-F*, 5′-CAGCCTGGACTTCGCGAG-3′; *INTU promoter-R*, 5′-TGAAGGCGGTGGTGTCAG-3′; *IFT88 promoter-F*, 5′-AAAACGGACACCTTAAGCGC-3′ and *IFT88 promoter-R*, 5′-CTTGTGAACCTTGGAAGCCC-3′.

### RNA extraction, reverse transcription and real-time PCR

The total RNA was isolated from cultured cells using TRIzol™ reagent (15596018, Thermo Fisher Scientific). The reverse transcription was performed using ImProm-II™ Reverse Transcription System (A3803, Promega) and random hexamer (N8080127, Thermo Fisher Scientific) according to the manufacturers’ instructions. Quantitative real-time PCR was performed using SYBR™ Green PCR Master Mix (4309155, Thermo Fisher Scientific) on the Bio-Rad CFX96 system. Relative gene expression was determined via normalizing against *β-ACTIN* using the 2^−ΔΔCT^ method. Primers used in this study were *INTU-F*, 5′-CGCATAGATGAACGGCTAGC-3′; *INTU-R*, 5′-AGCGTTCTTCTGCATGTTGG-3′; *IFT88-F*, 5′-CTGCAACCAATCTCTCAGCC-3′; *IFT88-R*, 5′-GCGGCCTTCTCATAATCACC-3′; *MALAT1-F*, 5′-ATGCGAGTTGTTCTCCGTCT-3′; *MALAT1-R*, 5′-TATCTGCGGTTTCCTCAAGC-3′; *β-ACTIN-F*, 5′-ATGTGCAAGGCCGGTTTCGC-3′ and *β-ACTIN-R*, 5′-CGACACGCAGCTCATTGTAG-3′.

### Immunoblotting

Protein samples were harvested from cells using the SDS sample buffer (100 mM Tris-HCl, pH 6.8, 2% SDS, 40% glycerol, 5% β-mercaptoethanol, and 0.1% bromophenol blue). Samples were heated at 99°C for 10 min prior to being subjected to the immunoblot analysis. The protein samples were then transferred to a PVDF membrane (IPVH00010, pore size 0.45 μm, Merck Millipore). The membrane was blocked using 5% nonfat milk at 25°C for 1 h, followed by the incubation of primary antibodies at 4°C for 16 h. Primary antibodies used were anti-TCF4 (ab217668, 1:1,000, abcam), anti-INTU (ab229243, 1:1,000, abcam), anti-IFT88 (13967-1-AP, 1:1,000, Proteintech) and anti-β-TUBULIN (ab6046, 1:2,000, abcam). The membrane was washed three times with 1× TBST each for 10 min, before being subjected to the incubation of secondary antibodies at 25°C for 1 h. Secondary antibodies used were HRP-conjugated goat anti-rabbit IgG (H + L) (11-035-045, 1:5,000) and HRP-conjugated goat anti-mouse IgG (H + L) (115-035-062, 1:10,000) from Jackson ImmunoResearch. The membrane was washed three times with 1× TBST each for 10 min, prior to the detection of chemiluminescent signal. The signal was developed using Immobilon Forte Western HRP substrate (WBLUF0100, Merck Millipore), and the images were captured and processed using ChemiDoc™ Touch Imaging System (170-8370, Bio-Rad).

### Statistical analysis

The two-tailed, unpaired Student’s *t* test was used for the comparison between two experimental groups. ^*^, ^**^ and ^***^ represent *p* < 0.05, *p* < 0.01 and *p* < 0.001, respectively, which are considered statistically significant. ns indicates no significant difference. GraphPad Prism version 9.0.0 was used for statistical analysis.

## Supplementary Materials

Supplementary Figures

Supplementary Table 1

Supplementary Table 2
