# Supplementary material for: Multifaceted investigation underlies diverse mechanisms contributing to the downregulation of Hedgehog pathway-associated genes INTU and IFT88 in lung adenocarcinoma and uterine corpus endometrial carcinoma
Source: Aging (Albany NY). 2022 Sep 7;14(19):7794–823. doi: 10.18632/aging.204262 (PMC9596204; doi:10.18632/aging.204262)
Supplement: Supplementary Figures [file aging-14-204262-s001.pdf]

## SUPPLEMENTARY FIGURES

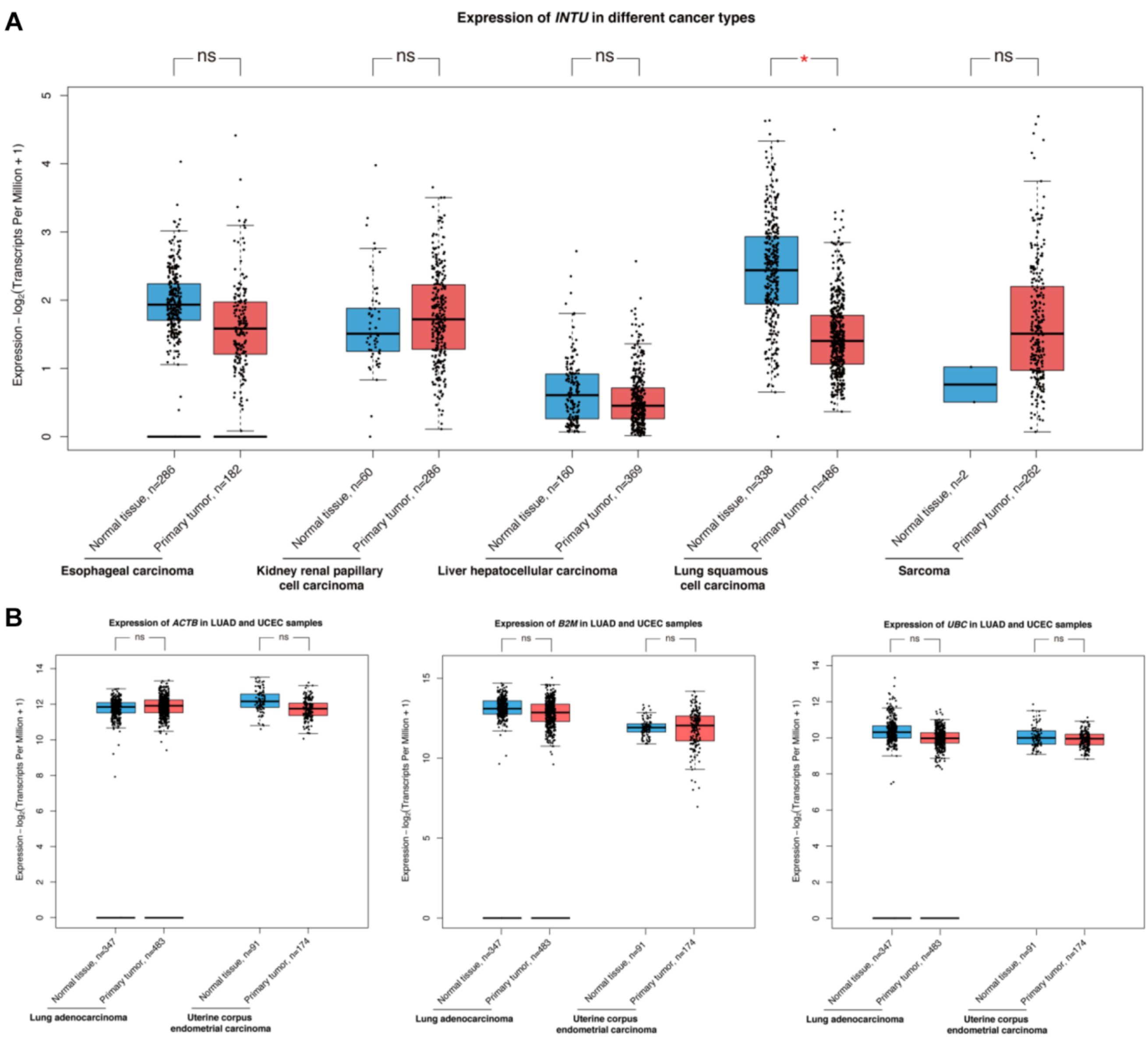


**Supplementary Figure 1. Evaluation of *INTU* and housekeeping gene levels in different cancer types.** (**A**) The expression of *INTU* was significantly downregulated in LUSC tumor samples. The *INTU* expression was not significantly altered in ESCA, KIRP, LIHC and SARC tumor samples when compared to their respective normal control samples. (**B**) The expression of housekeeping genes *ACTB*, *B2M* and *UBC* was not altered in LUAD and UCEC tumor samples when compared to their respective normal control samples.

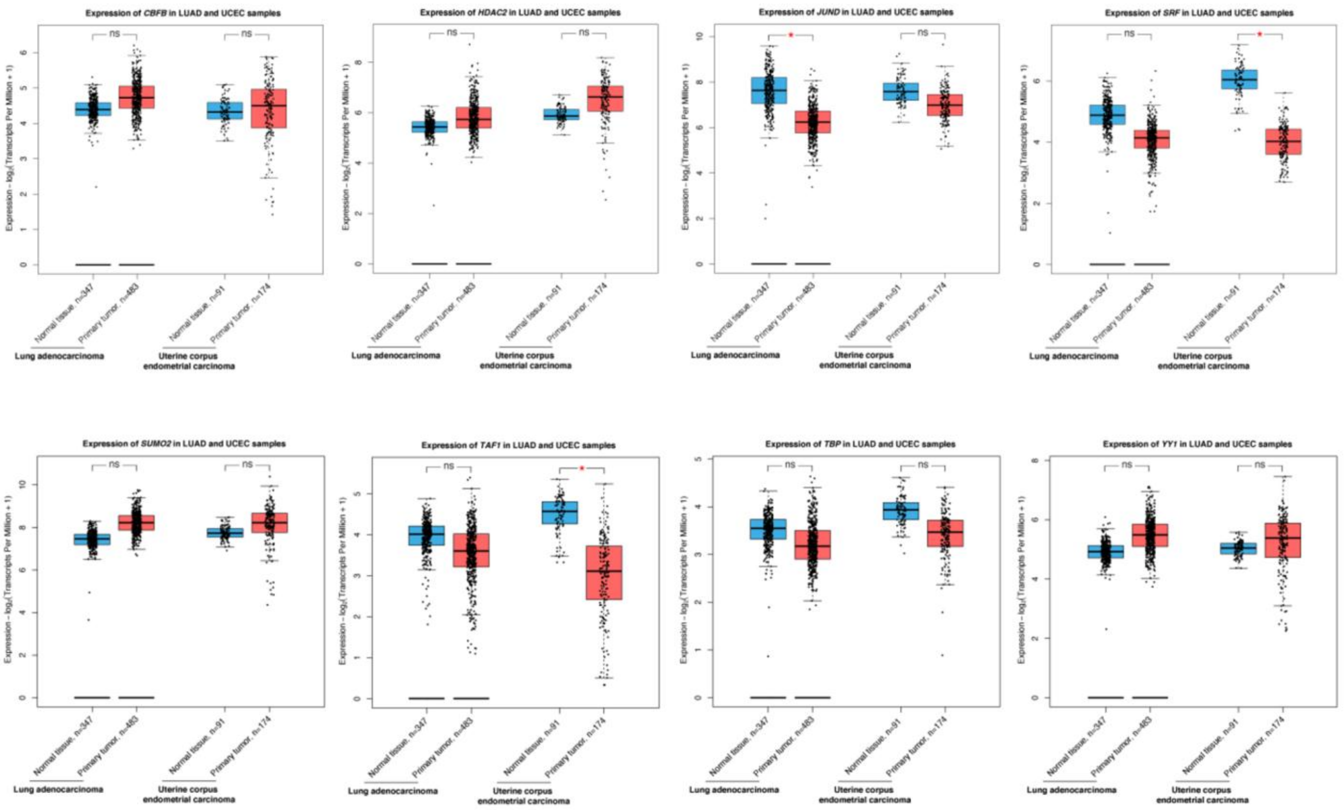


**Supplementary Figure 2. Examination of *CBFB*, *HDAC2*, *JUND*, *SRF*, *SUMO2*, *TAF1*, *TBP* and *YY1* levels in LUAD and UCEC samples.** None of the transcription factors examined showed significant change of expression in both LUAD and UCEC tumor samples.

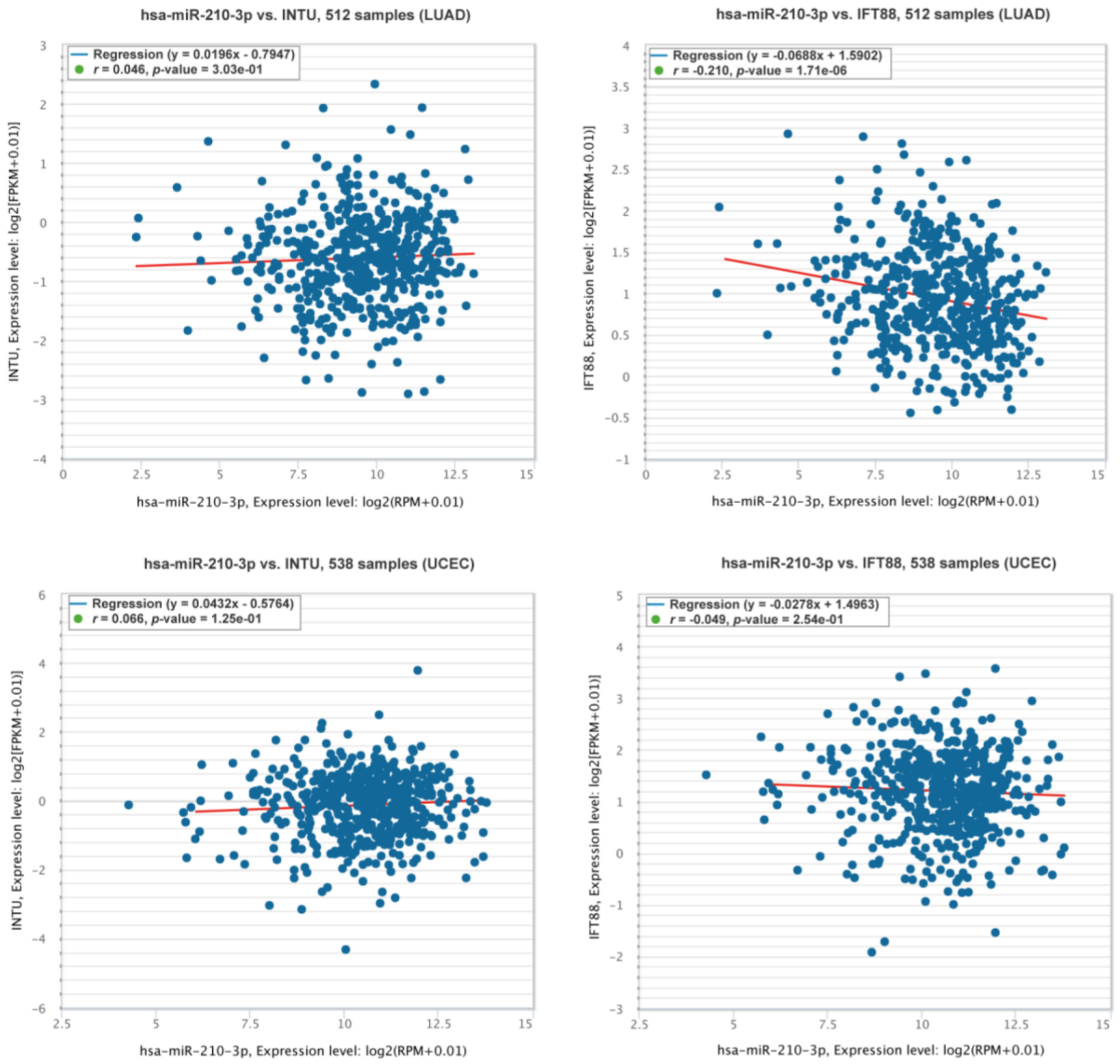


**Supplementary Figure 3. Evaluation of the correlation between *hsa-miR-210-3p* expression and *INTU* and *IFT88* levels in LUAD and UCEC tumor samples.** No significant correlation was detected between the expression of *hsa-miR-210-3p* and mRNA levels of *INTU* and *IFT88* in LUAD and UCEC tumor samples, except for in LUAD tumor samples, expression of *hsa-miR-210-3p* negatively correlated with *IFT88* mRNA level.

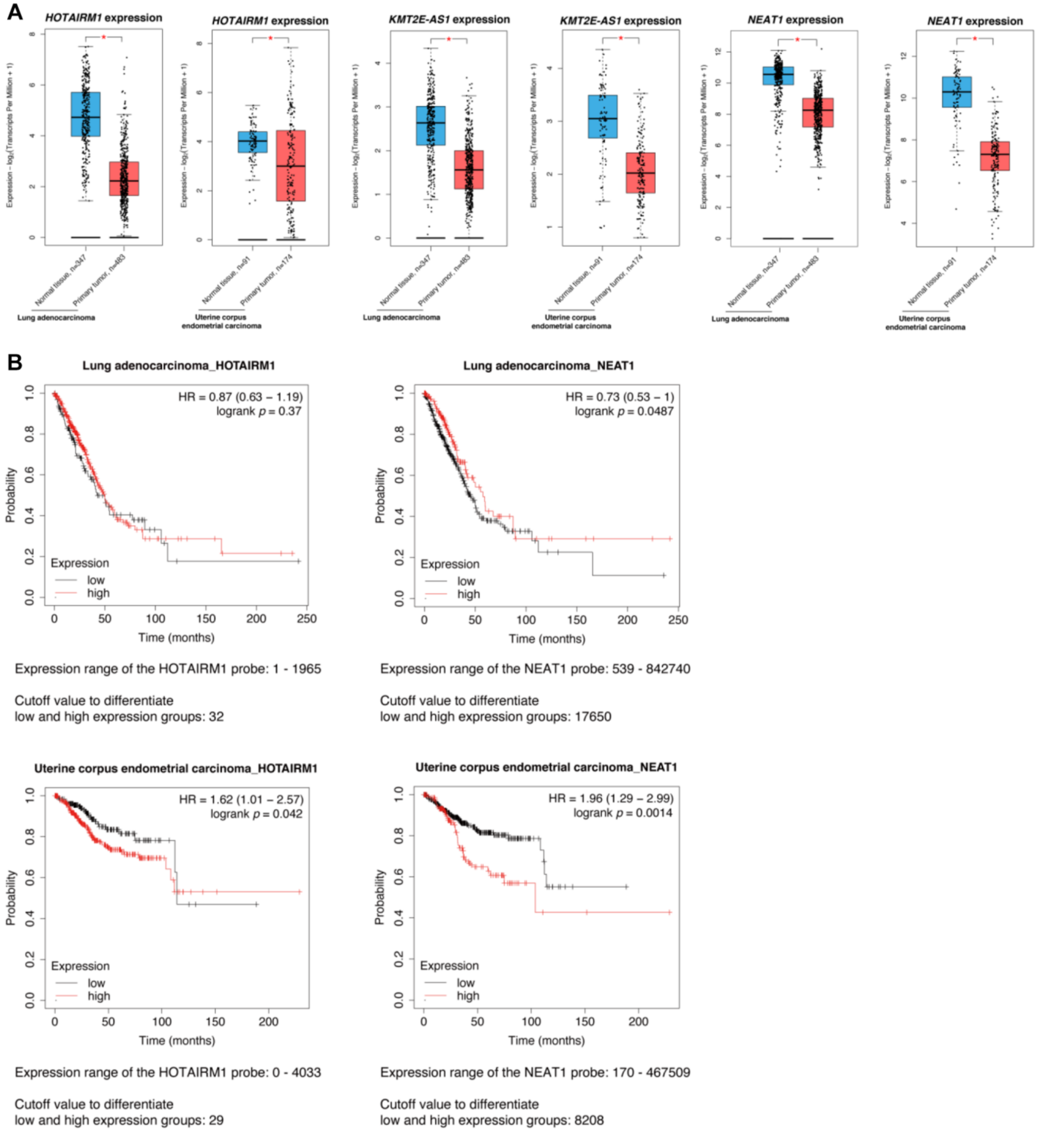


**Supplementary Figure 4. Evaluation of the expression and prognostic significance of lncRNAs in LUAD and UCEC tumor samples.** (**A**) The expression of *HOTAIRM1*, *KMT2E-AS1* and *NEAT1* was significantly downregulated in LUAD and UCEC tumor samples. (**B**) The LUAD patients with decreased *NEAT1* level showed reduced survival probabilities. Higher levels of *HOTAIRM1* and *NEAT1* were found associated with poor survival probabilities in UCEC patients. No correlation between *HOTAIRM1* level and OS probabilities was detected in LUAD patients.

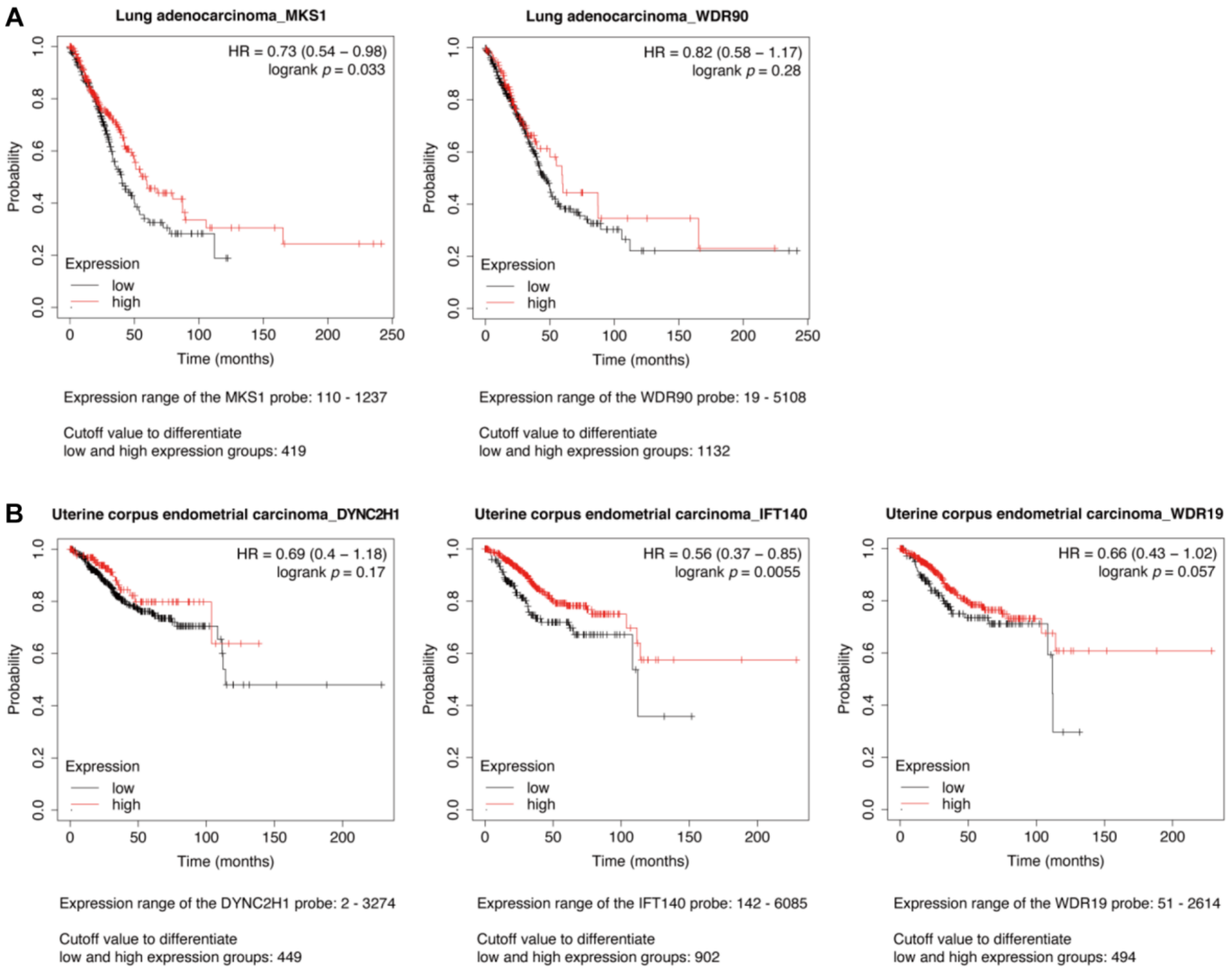


**Supplementary Figure 5. Evaluation of the prognostic significance of enriched Hh-related genes in LUAD and UCEC patients.** (**A**) Decreased level of *MKS1* correlated with poor OS probabilities in LUAD patients, whilst the *WDR90* level didn't show a significant correlation with OS probabilities in LUAD patients. (**B**) The UCEC patients with lowered *IFT140* level showed decreased OS probabilities. Neither *DYNC2H1* nor *WDR19* level significantly correlated with OS probabilities in UCEC patients.

A

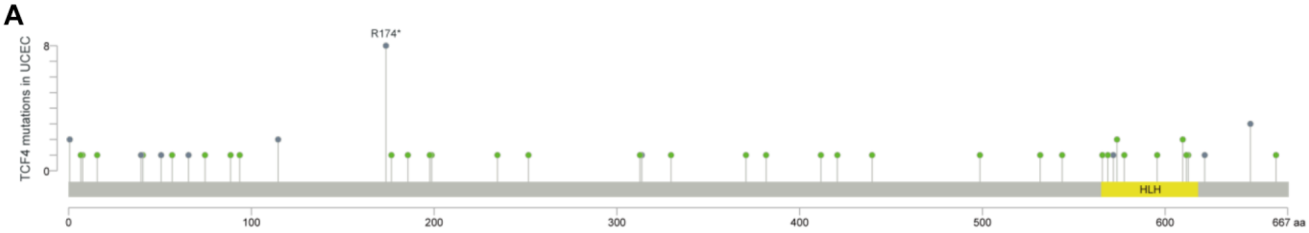


B

| Cancer type | Sample ID | TCF4 mutation | | | Functional Impacts | |
|---|---|---|---|---|---|---|
| | | Amino acid change | HGVSc | Mutation type | SIFT | MutationAssessor |
| Uterine Corpus Endometrial Carcinoma | TCGA-AP-A056-01 | R174* | ENST00000356073.4:c.520C>T | Nonsense | N/A | N/A |
| Uterine Corpus Endometrial Carcinoma | TCGA-AX-A05Z-01 | | | | | |
| Uterine Corpus Endometrial Carcinoma | TCGA-B5-A0JY-01 | | | | | |
| Uterine Corpus Endometrial Carcinoma | TCGA-B5-A11E-01 | | | | | |
| Uterine Corpus Endometrial Carcinoma | TCGA-B5-A1MR-01 | | | | | |
| Uterine Corpus Endometrial Carcinoma | TCGA-BS-A0UF-01 | | | | | |
| Uterine Corpus Endometrial Carcinoma | TCGA-EO-A3AV-01 | | | | | |
| Uterine Corpus Endometrial Carcinoma | TCGA-QF-A5YS-01 | | | | | |

C

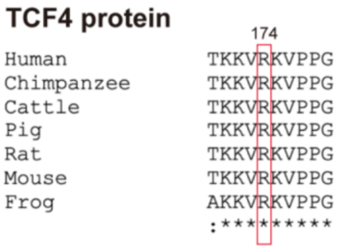


**Supplementary Figure 6. Illustration of the mutations in TCF4 protein from LUAD and UCEC tumor samples.** (**A**) A relative higher mutation frequency was identified at TCF4$^{R174}$ residue from UCEC tumor samples. (**B**) The detailed mutation site of TCF4$^{R174}$ mutant protein from UCEC tumor samples. (**C**) The TCF4$^{R174}$ residue was highly conserved among different species.

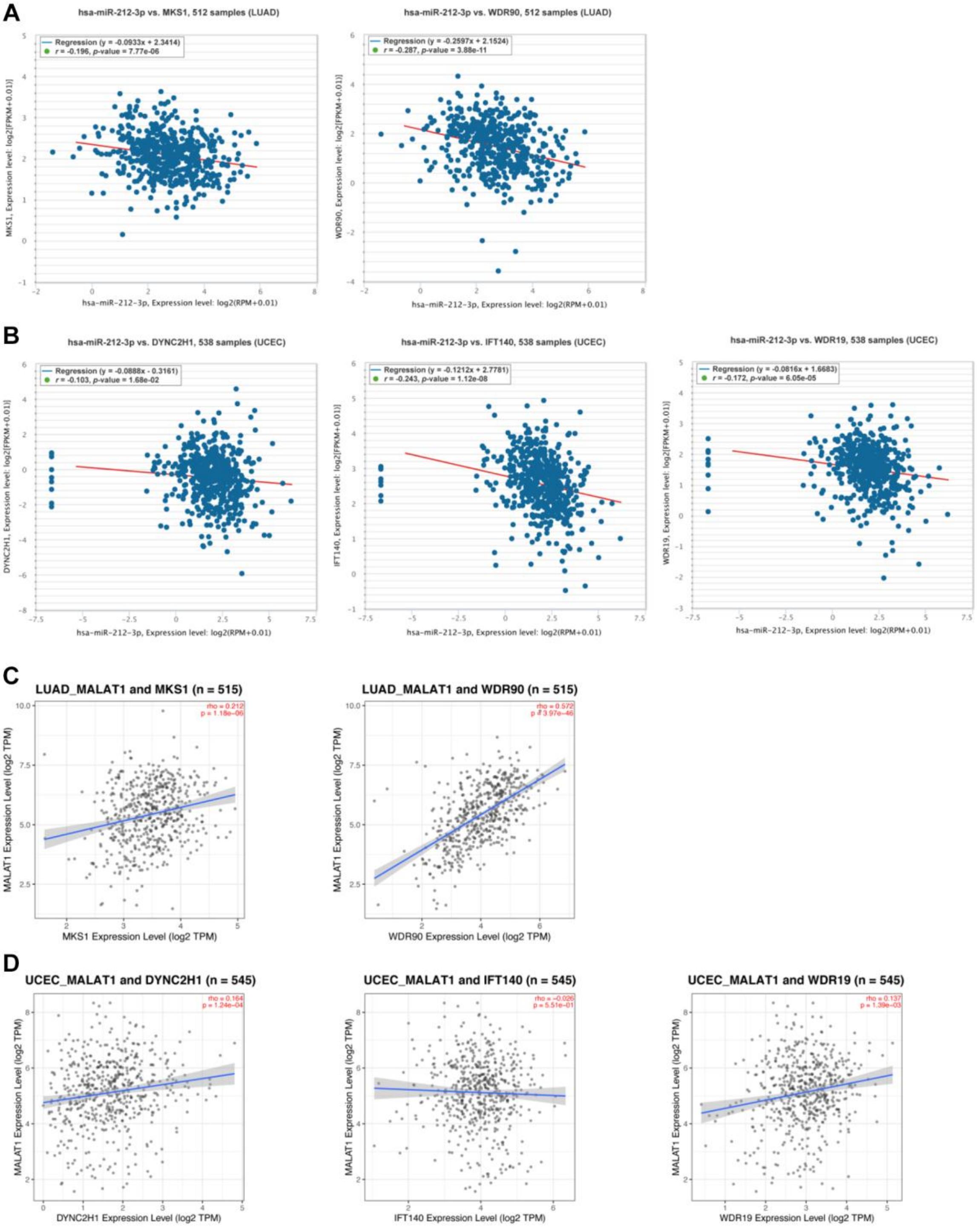


**Supplementary Figure 7. The *MALAT1-hsa-miR-212-3p* signaling axis regulates enriched Hh-related genes in LUAD and UCEC samples.** (**A**) The expression of *hsa-miR-212-3p* was found negatively associated with the mRNA levels of *MKS1* and *WDR90* in LUAD samples. (**B**) Negative correlation was determined between *hsa-miR-212-3p* expression and mRNA levels of *DYNC2H1*, *IFT140* and *WDR19* in UCEC samples. (**C**) The expression of *MALAT1* positively correlated with the levels of *MKS1* and *WDR90* in LUAD samples. (**D**) The expression of *MALAT1* positively correlated with the levels of *DYNC2H1* and *WDR19* in UCEC samples. No significant correlation was determined between *MALAT1* and *IFT140*.
