# Supplementary material for: Multifaceted investigation underlies diverse mechanisms contributing to the downregulation of Hedgehog pathway-associated genes INTU and IFT88 in lung adenocarcinoma and uterine corpus endometrial carcinoma
Source: Aging (Albany NY). 2022 Sep 7;14(19):7794–823. doi: 10.18632/aging.204262 (PMC9596204; doi:10.18632/aging.204262)
Supplement: Supplementary Table 2 [file aging-14-204262-s003.pdf]

SUPPLEMENTARY TABLE

Supplementary Table 2. The promoter sequences of *INTU* and *IFT88*.

***INTU* gene promoter sequence (*INTU*^{CpG} sequence is in blue and TCF4 binding site is highlighted):**
ATCATTAATCTGTAATCTATAACCTATGATAGCTCACATTTTAAACTATTACGCTCCAGTTTCTCCATTTATTCTCC<br>TTCAGTGGTTCCCCTTACCATTCTGGCCTGTTCTGACTTAGGGACAGTCTACAGTAGGAAGTCACACACGCGTCA<br>CTTTTCCCACGATGGAAAAACCACCAAGCTAATTTTGTCTTCTCTTTGACCACAGGCCATAGAATAGTTCACTGA<br>AATACCTAATGCCCTAGAGTAGAGACTGTCTCCTGGGGTCAAGTATATTTTAAAGCAAATAAATCCCCCCAAAA<br>GAGAATAAAGCCACATTAGACAAGTCAGAGTCCACCTTTTATTCACTCTTGTACCTCCAAGGACTAGAACTCGGC<br>CTGGCACATAGCAAGTGGTAATACACATTTGCACGGACGGATGAATGTATATGGCTTCTTTAGGCTGAATTAAA<br>ACTCCCACCAAGAGCAGACAACTACCTTGCCTCTTCCCCACTCGTTTTCGGGTCTTCCACAGAGCAGCCAGAGCC<br>TCAGAGGCCCTTGAGAGTTTCCTCCACTCCTCCCTTGTTTGCAGCGCTAGAAGCTGCAGGTGGTAGTTCCTACAC<br>TGGGGGCGGCGCCTGGACGCGGGTGTCCCTGGCCAAGGCGGCCTCGCTGTCCTGGAAGGGAGGGTGAAGAGCT<br>GCATCCCGCACTAGGCGGCGAAAGAGGGCAGCGCCAAGCGGCGGGGTCCGGAGGCGCTCGACGGCTCGCGCCC<br>AGCGCCGGAGACGGGCTGTGTGTTGGGCCAGTGGAAGACACCGGAGAAACCCAGACGTGGAAGACCGGGCAGC<br>CTGGACTTCGCGAGCCCTGGTGGGGCTGGCGGCCCACAGAGCCCCCACCTGCCCCGAGCTCCCACAGCGAGGAG<br>TGGCCGCGCCGCCCGCCAGTGCGCCGGGCTCCGAGACCGGCAGGGGAGCACGCGGGCGAAGGAGGGGCCGCCG<br>TCGCTGACACCACCGCCTTCAGCCCTTGGCTTCCGCGCGTCGGAGGCTGGCACCTCCAGGTTCACCGCGGCGGCC<br>GGAGCTGTGCGGGGGCCAGACGGTTCGGCGGGAGCCGGGGCTGGGACCTGGGTGACCTGTCGTCCGCCCCTGTA<br>GCGAGTCTCCAGTGGGCATGTTTCAGGTGGGCAGGTCCAGCATCCCCAAACCTGCCCCCCGCAGCCTGGAGGAC<br>CTGGACTCAGTGCAGCGTGTCCTGTTACACAG.
***IFT88* gene promoter sequence (*IFT88*^{CpG} sequence is in blue and TCF4 binding site is highlighted):**
GCTGGTCTCCAACTCCTCACCTCAGGTGATCCGCCAGCCTCAGCCTCCCAAAGTGCTGGGATTATAGGCATGAGC<br>CACTGCGCCCGGCCAACACTACTTATTTTATAAAAACTCATTAATTACTGGTGTCTTTAGACTCCCTCTGTCCCTC<br>CCCTCCATCCCCAACATTTACTTTTAAAATTTAAGACAAAACTTAAACATTAAACAATTATGAATTTAATTCCCCC<br>ATTTATATTACATTGATTAAGCTTTACACTATGACCCAGGTTCTAGGTGGTTGGGACACAATGGTTCAATCCCTGT<br>CTAGTAAATAAAACGGACACCTTAAGCGCTACAATCAAGGTGCGCACGGGGTGCTACAGAACAATACGCAGAC<br>AGGAGGAGGTGGGGGGAAAGGTGAGACTTCCCAGGCAGGTGTCATCCCAGAATAACTTTTACACAGGGTGACT<br>AAGCGAGTTAGTGACTGCGCGGAAAACGGGCTTCCAAGGTTCACAAGGCTCGTGCTGCGGCTCCGGGAGTTATG<br>TCACAGTAAGCTTACTATCATCCTTTGGGCATCTGCTTTACGGATGAGTTCATCAGGATTTAAAGGATCTTGGTTC<br>CATATCCTTCCCCTTCCTCACAGAGGCCGCCAGCCCGGAGCCCCTCTAGGCCCTCCTCCCTCCTGCATCTACTGGC<br>CGCGAGCCTTTCCCTCCCCGCCCCCTTCACACAGGCCGCCCCCAGCCTCCCAACCCCCCGGTTCCGTTCCACGGG<br>AGGCCCCGGCCTCCCTGCCCTCTCCTCCACCGTTCTACCCGCATCGCCCGGCTTCCCGCAAGCCGCTGGCACCGT<br>CCCCTCAACACCCTCCGCCCACCGCCACTCCCTTCCACTGAGGGGGACCGGGCTGCCTTCTCTCTGACCCGCCGT<br>TCTCCGCCCCCACCCACTTCCCCAGGCCTCCCGCACCACCCCCTTCCCGCCGCGCTCTCTCCCGCCCCCGCGCTTC<br>CGGCCCCGCCCGCCCCCGCGCGGAGGACTGTGGGAGCGGCTTCCTTGGATTCCGCGCTTGGCAACGGCTCGGCG<br>TCGCGCTTTGGCCAACCGCTGCGTCGTCCCTGGGCCCGAATAACTGTCGCCCGCTTCCCTCAGCGTGAG.
